# Anti-Inflammatory Activity of Monosubstituted Xestoquinone Analogues from the Marine Sponge *Neopetrosia compacta*

**DOI:** 10.3390/antiox11040607

**Published:** 2022-03-22

**Authors:** Shalice R. Susana, Lilibeth A. Salvador-Reyes

**Affiliations:** Marine Science Institute, University of the Philippines—Diliman, Velasquez St., UP Diliman, Quezon City 1101, Philippines; shalicesusana@msi.upd.edu.ph

**Keywords:** marine sponge, xestoquinone, anti-inflammatory, Nrf2 activation, natural products

## Abstract

Chronic inflammation is recognized as a contributor to multiple chronic diseases, such as cancer, cardiovascular, and autoimmune disorders. Here, a natural products-initiated discovery of anti-inflammatory agents from marine sponges was undertaken. From the screening of 231 crude extracts, a total of 30 extracts showed anti-inflammatory activity with no direct cytotoxic effects at 50 μg/mL on RAW 264.7 (ATCC^®^TIB-71™) murine macrophage cells stimulated with 1 μg/mL lipopolysaccharide (LPS). Bioactivity-guided purification of the anti-inflammatory extract from the sponge *Neopetrosia compacta* led to the isolation of xestoquinone (**1**), adociaquinone B (**2**), adociaquinone A (**3**), 14-hydroxymethylxestoquinone (**4**), 15-hydroxymethylxestoquinone (**5**), and an inseparable 2:1 mixture of 14-methoxyxestoquinone and 15-methoxyxestoquinone (**6**). Compounds **1**–**6** caused a concentration-dependent reduction of nitric oxide (NO) production in LPS-stimulated RAW 264.7 cells, with **4**–**6** having low micromolar IC_50_ and acceptable selectivity index. Gene expression analysis using qRT-PCR showed that **1**, **5**, and **6** downregulated *Il1b* and *Nos2* expression by 2.1- to 14.8-fold relative to the solvent control at 10 μM. Xestoquinone (**1**) and monosubstituted analogues (**4**–**6**), but not the disubstituted adociaquinones (**2** and **3**), caused Nrf2 activation in a luciferase reporter MCF7 stable cells. Compounds **5** and **6** caused a modest increase in *Nqo1* gene expression at 10 μM. The anti-inflammatory activity of xestoquinone (**1**) and monosubstituted analogues (**4**–**6**) may, in part, be mediated by Nrf2 activation, leading to attenuation of inflammatory mediators such as IL-1β and NOS2.

## 1. Introduction

Marine organisms are prolific sources of clinically relevant secondary metabolites with unique chemistry and a broad range of biological activities [1,2]. Sponges (Porifera) are among the richest source of marine natural products, with ≈5000 compounds isolated, equivalent to ≈30% of identified marine-sourced compounds [3,4]. Marine sponges, being sessile invertebrates, partly utilize small molecule compounds for defense, competition, and adaptation [1,5,6].

The increasing evidence linking chronic inflammation to several other diseases warrants the discovery and development of anti-inflammatory drugs [7,8,9]. From 2009–2017, more than 25 sponge-sourced compounds have been identified as anti-inflammatory leads [10,11,12,13], with manoalide, contignasterol, scalaradial, and their derivatives reaching clinical trials. Manoalide and scalaradial are potent inhibitors of phospholipase A2 (PLA2) that inhibit the biosynthesis of inflammatory lipids such as prostaglandins [7,14,15]. Contignasterol exhibits anti-inflammatory effects by attenuating histamine release in anti-immunoglobulin E (Anti-IgE)-stimulated leukocytes [7,14,15]. Hence, it is evident that anti-inflammatory agents from marine sponges are structurally diverse with distinct target receptors and mechanisms of action.

In this study, we undertook the discovery of anti-inflammatory compounds from marine sponges from the Philippines. We screened a total of 231 sponge extracts for anti-inflammatory activity on LPS-stimulated RAW 264.7 murine macrophage cells. Bioactivity-guided purification afforded six compounds from the sponge *Neopetrosia compacta*. The mechanism of anti-inflammatory activity was evaluated using a luciferase reporter assay and differential gene expression analysis.

## 2. Materials and Methods

### 2.1. General Experimental Procedures

RAW 264.7 murine macrophage cells (ATCC^®^TIB-71™) and Nrf2-luciferase reporter MCF7 stable cells (SL-0010) were purchased from American Type Culture Collection (ATCC, Mannasas, VA, USA) and Signosis, Inc. (Santa Clara, CA, USA), respectively. Solid-phase extraction (SPE) was carried out using pre-packed Strata^®^ SPE-C18 (1 g/6 mL, 55 µm) cartridges (Phenomenex, Torrance, CA, USA). Reversed-phase high performance liquid chromatography (RP-HPLC) was performed using Shimadzu Prominence LC-20AT equipped with an SPD-M20A photo-diode array (PDA) detector and Phenomenex Synergi Hydro-RP column (4 µm, 80 Å, 250 × 10 mm). Low resolution mass analysis was carried out on a Shimadzu LCMS-8040 triple quadrupole mass spectrometer equipped with electrospray ionization (ESI) ion source detector using a linear gradient 5–100% aq. CH_3_CN with 0.1% (*v*/*v*) formic acid. High resolution mass analysis was carried out on Waters Xevo^®^ G2-XS Quadrupole Time-of-Flight (QToF) mass spectrometer equipped with ESI ion source detector using a linear gradient 5–100% aq. CH_3_CN with 0.1% (*v*/*v*) formic acid. The 1D- and 2D-NMR spectra were recorded on a Varian 500 MHz NMR equipped with a 3 mm probe. Statistical analyses were carried using GraphPad Prism version 9.3.1 for Windows, GraphPad Prism Software (San Diego, CA, USA).

### 2.2. Extraction of Sponge Samples

Sponge samples were sequentially extracted with three solvents of different polarities: *n*-hexane, 4:1 CH_2_Cl_2_:MeOH, and MeOH. Samples were macerated and extracted with *n*-hexane. After 24 h, the filtrate was collected and dried *in vacuo* to yield the *n*-hexane soluble extract. The sponge biomass was further soaked in 4:1 CH_2_Cl_2_:MeOH, and the filtrate was collected and dried to yield the CH_2_Cl_2_:MeOH extract. The same procedure was done to obtain the MeOH extract. A total of 77 marine sponges were extracted to generate 231 crude extracts.

### 2.3. Nitric Oxide Production Assay

The anti-inflammatory activity of the extracts and compounds was assessed according to the procedure of Ratnayake et al. (2013) [16]. RAW 264.7 murine macrophage cells (passage number ≤ 6) were grown in Dulbecco’s Modified Eagle’s Medium (DMEM) supplemented with 10% fetal bovine serum (FBS) and 1% antibiotic-antimycotic solution. At 24 h post-incubation, the cells (200,000 cells/mL) were treated with extracts (50 and 5 µg/mL) or pure compounds in triplicate. For pure compounds, a 20 mM stock was prepared in DMSO and serially diluted to generate eight concentrations ranging from 0.032–100 µM. DMSO (0.5%) served as the solvent control. Dexamethasone (50 µM) and *t*BHQ (10 µM) served as positive controls. LPS (1 µg/mL) was added to each well after 1 h of extract or compound pre-treatment. A reverse treatment was also done for compounds, where cells were first pre-treated with LPS (1 µg/mL) prior to the addition of the compound.

After 24 h of incubation, NO production was assessed by measuring the nitrite concentration using the Griess Reagent System (Promega, Madison, WI, USA) according to the manufacturer’s protocol. Results were presented as %NO production relative to LPS-stimulated solvent control. A concentration-dependent response curve for NO production of the pure compounds was generated, and the IC_50_ was determined using a non-linear regression, inhibitor vs. normalized response—variable slope analysis. Samples were tested in at least one independent experiment performed in triplicate.

### 2.4. Tetrazolium-Based Cell Viability Assay

MTT solution (5 mg/mL in PBS, 15 µL) was added to the original assay plate, and the reaction was terminated with the addition of acidified sodium dodecyl sulfate (SDS) solution after 2 h. Absorbance was measured at 570 nm. Results are presented as % cell viability relative to the solvent control. The concentration-dependent response curve for cell viability of the pure compounds was generated, and the IC_50_ was determined using a non-linear regression, inhibitor vs. normalized response—variable slope analysis. Samples were tested in at least one independent experiment performed in triplicate.

Statistical comparison of the cell viability IC_50_ of **2**–**6** relative to xestoquinone (**1**) was analyzed using one-way ANOVA and Tukey’s post-hoc test at *p*-value < 0.05. Comparison of the IC_50_ values from NO production and cytotoxicity assessment for each of the compounds were analyzed using a one-tailed unpaired *t*-test at *p*-value < 0.05.

### 2.5. Bioactivity-Guided Purification of N. compacta (Lala1F)

The source sponge was previously identified as *Neopetrosia compacta* [17]. The CH_2_Cl_2_:MeOH soluble extract from *N. compacta* (sponge code: Lala1F.d, 3.3496 g) was fractionated using pre-packed C18-SPE column with an increasing gradient of MeOH in H_2_O (25%, 50%, and 100%). The bioactive fraction eluting with 100% MeOH (494 mg) was subjected to RP-HPLC (Phenomenex; Synergi Hydro-RP, 250 × 10 mm, 4 µm; flow rate, 2.0 mL/min) with H_2_O and CH_3_CN as mobile phase, with a gradient consisting of isocratic 40% CH_3_CN for 5 min, 40–100% CH_3_CN for 30 min, and 100% CH_3_CN for 10 min. Peaks were collected based on the UV absorbance monitored at 220, 254, and 360 nm to afford 13 HPLC fractions. HPLC fractions 4 and 5 yielded adociaquinones B (**2**, *t*_R_ = 18.8 min, 11.8 mg) and A (**3**, *t*_R_ = 19.9 min, 6.5 mg), respectively. The 14-hydroxymethylxestoquinone (**4**, 3.6 mg) and 15-hydroxymethylxestoquinone (**5**, 2.0 mg) were purified from the fractions with *t*_R_ = 24.4 min and 26.1 min, respectively. The fraction with *t*_R_ = 30.5 min gave a 2:1 mixture of 14- and 15-methoxyxestoquinone (**6**, 3.8 mg). The fraction at *t*_R_ = 32.0 min was further purified using RP-HPLC (Phenomenex; Synergi Hydro-RP, 250 × 10 mm, 4 µm; flow rate, 2.0 mL/min) with H_2_O and CH_3_CN as mobile phase, with a gradient consisting of 70% CH_3_CN for 5 min, 70–100% CH_3_CN for 15 min, and 100% CH_3_CN for 5 min to give xestoquinone (**1**, 18.1 mg, *t*_R_ = 16.3 min). Purified compounds were subjected to MS and NMR analyses for structure elucidation. The spectroscopic data for **1**–**6** are provided in the Supplementary Materials, Figures S2–S31.

### 2.6. RNA Extraction, Quantitative RT-PCR, and Differential Expression Analysis

RAW 264.7 cells (passage number ≤ 6) were seeded in a 6-well microplate at a density of 2.5 × 10^5^ cells/well and incubated for 24 h at 37 °C with 5% CO_2_ prior to treatment. Cells were treated with 10 μM compound or 0.5% DMSO in duplicate and incubated for 1 h before stimulation with LPS (1 µg/mL). At 12 h post-treatment, total RNA was extracted with Qiagen RNeasy extraction kit (Germantown, MD, USA) according to manufacturer’s protocol. RNA was reverse-transcribed from 2 µg of total RNA using SuperScript IV Reverse Transcriptase (Invitrogen, Waltham, MA, USA), 1 mM dNTP, and Oligo (dT)_12–18_ primer (0.5 µg/mL, Invitrogen). Quantitative real time-PCR (qRT-PCR) was performed in ABI 7500 Fast Real-Time PCR (Applied Biosystems, Bedford, MA, USA). qRT-PCR was performed as a 25 µL total reaction mixture, composed of 1X TaqMan^®^ universal master mix, 1X TaqMan^®^ gene expression assay mix, 2 µL of cDNA, and 9.25 µL RNase-free sterile water. Analyses were carried out using probes for *Il1b* (Assay ID Mm00434228_m1), *Nos2* (Mm00440502_m1), and *Nqo1* (Mm01253561_m1). The thermocycler program was designed as follows: 50 °C for 2 min, 95 °C for 10 min, and 40 cycles of 95 °C for 15 s and 60 °C for 1 min. Mouse *Gapdh* (Mm99999915_g1) was used as the internal control for normalization. The measurement of gene expression is reported in cycles to threshold (Ct) PCR, whose value represents the cycle number at which the amount of amplified DNA of the target gene reached the threshold level. The relative gene expression was analyzed using the comparative Ct method to compare the Ct value of the target gene to the internal control in a treatment sample. Statistical analysis on the relative gene expression was carried out using one-way ANOVA and Tukey’s multiple comparison test with *p*-value < 0.05. Analysis was done in two independent experiments with three technical replicates each.

### 2.7. Direct Antioxidant Activity Assay

The direct antioxidant activity of **1**–**6** was determined using the 2,2-diphenyl-1-picrylhydrazyl (DPPH) scavenging method. Half-log dilution of **1**–**6** (0.032 to 100 µM) was prepared in MeOH. Then, 20 μL of 1 mM DPPH was dispensed into each well and incubated for 30 min at room temperature with shaking. Absorbance was measured at 517 nm, and results are presented as % radical scavenging activity (% RSA) relative to the solvent control. Ascorbic acid (0.032–100 µM) and *t*BHQ (0.032–100 µM) served as positive controls, while 0.5% DMSO served as solvent control. The analysis was done in triplicate.

### 2.8. Nrf2-ARE Activation Luciferase Reporter Assay

Nrf2-luciferase reporter MCF7 stable cells (Signosis, Inc.) were grown in high glucose DMEM (Hyclone, Logan, UT, USA) supplemented with 10% FBS, 1% antibiotic-antimycotic solution, and 75 µg/mL geneticin. A 100-µL cell suspension was plated with a seeding density of 20,000 cells/well. The plate was incubated at 37 °C with 5% CO_2_ for 24 h prior to treatment. Cells were treated with compounds at half-log dilution, ranging from 0.032–100 µM (0.5% DMSO). DMSO (0.5%) and *t*BHQ (0.01–32 μM) served as solvent and positive controls, respectively. After 8-, 12-, and 16-h incubation, the medium was removed, and cells were washed with 100 µL 1X PBS. Lysis buffer (Signosis, Inc.) was added, and the plate was further incubated for 15 min at room temperature. A 100-µL firefly luciferase substrate (Signosis, Inc.) was added, and luminescence was measured. Nrf2 fold activation of treated cells was assessed relative to the solvent control. The assay was done in two independent experiments in triplicate.

For the cytotoxicity counter-screen, Nrf2-luciferase reporter MCF7 stable cells were grown, seeded, and treated identically with the reporter assay. Cell viability was done using the tetrazolium reagent, as indicated previously.

## 3. Results

### 3.1. Screening for Anti-Inflammatory Activity of Sponge Crude Extracts

**Figure 1:**
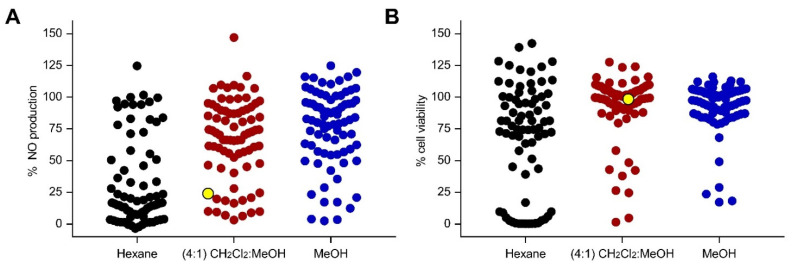
Screening for anti-inflammatory activity of 231 marine sponge crude extracts based on (**A**) NO production and (**B**) cell viability counter-screen at 50 µg/mL in LPS-stimulated RAW 264.7 cells. Each dot represents the % NO production (**A**) or % cell viability (**B**) of LPS-stimulated RAW 264.7 cells in response to the extract treatment. Lipophilic extracts are shown in black, while semipolar and polar extracts are displayed in maroon and blue, respectively. The CH_2_Cl_2_:MeOH (4:1) extract of *N. compacta* Lala1F sponge, highlighted in yellow, reduced the NO production by 76% at 50 µg/mL, with no observable cytotoxic effects. The positive controls, dexamethasone (50 µM) and *t*BHQ (10 µM), reduced NO production by 50% and 90%, respectively, with no observed cytotoxicity. Data are presented as the mean of % NO production or % cell viability (*n* = 3).

A total of 77 marine sponges were collected from two locations in the Philippines (Bolinao, Pangasinan, and Puerto Galera, Oriental Mindoro). Sponges were sequentially extracted with *n*-hexane, CH_2_Cl_2_:MeOH (4:1), and MeOH to yield 231 crude extracts. RAW 264.7 cells were pre-treated with the extracts 1 h prior to LPS stimulation. At a concentration of 50 µg/mL, a total of 30 extracts reduced NO production by ≥80%, with ≤20% decrease in cell viability in LPS-stimulated RAW 264.7 cells (Figure 1). Among the 30 anti-inflammatory extracts, 19 were hexane soluble, while 7 and 4 were CH_2_Cl_2_:MeOH and MeOH extracts, respectively. Screening at a lower concentration of 5 µg/mL yielded extracts that reduced the NO production by ≥80% with low cytotoxicity to LPS-stimulated RAW 264.7 cells (Figure S1). The CH_2_Cl_2_:MeOH extract of the sponge coded Lala1F reduced the NO production by 76% and 51% at 50 and 5 µg/mL, respectively, with no observable cytotoxic effects to LPS-stimulated RAW 264.7 cells (Figure 1, Table S1). This extract was pursued for bioactivity-guided purification. 

### 3.2. Bioactivity-Guided Purification of N. compacta Lala1F

**Figure 2:**
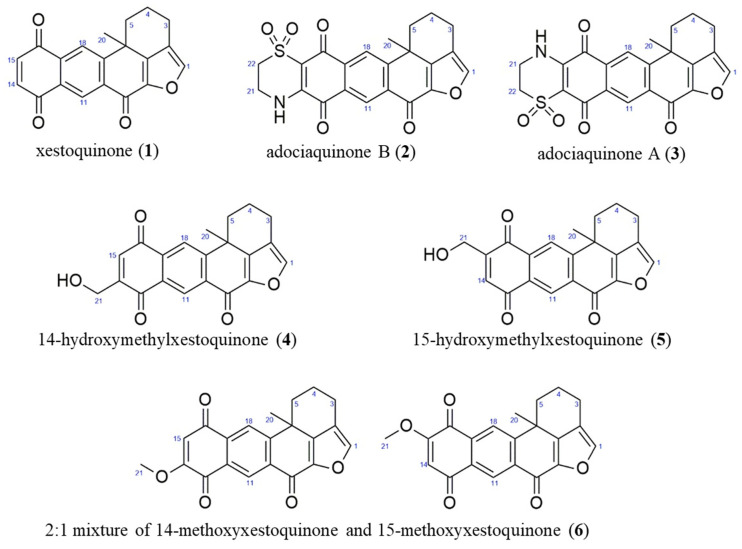
Anti-inflammatory compounds from the marine sponge *Neopetrosia compacta*.

The CH_2_Cl_2_:MeOH extract of *N. compacta* was subjected to bioactivity-guided purification. The ^1^H NMR spectra of **1**–**6** showed characteristic resonances for polycyclic polyketides belonging to the xestoquinone and halenaquinone family of compounds. The ^1^H NMR spectra of **2**–**6** displayed additional chemical shifts corresponding to methylene protons adjacent to the quinoid ring (in **2** and **3**), a hydroxymethyl moiety in **4** and **5**, and an -OCH_3_ group in **6**. Based on the MS and NMR spectral data of **1**–**6**, these were identified as xestoquinone (**1**), adociaquinone B (**2**), adociaquinone A (**3**), 14-hydroxymethylxestoquinone (**4**), 15-hydroxymethylxestoquinone (**5**), and an inseparable 2:1 mixture of 14-methoxyxestoquinone and 15-methoxyxestoquinone (**6**) (Figure 2), and agreed with reference literature values [18,19,20,21,22,23].

### 3.3. Biological Activity and Structure-Activity Relationship (SAR) Studies

RAW 264.7 cells were treated with half-log dilutions of compounds starting at 0.032 μM to assess the potency of the anti-inflammatory activity of **1**–**6** prior to LPS stimulation. A concentration-dependent reduction of NO production in LPS-stimulated RAW 264.7 cells was observed with treatments of **1**–**6**, with IC_50_ of 1.2–5.0 μM (Table 1). Structure-activity relationship (SAR) of xestoquinone (**1**) and analogues (**2**–**6**) showed that modifications in the quinone moiety did not influence the NO inhibitory activity of these compounds, indicating the importance of this moiety for the anti-inflammatory activity (Table 1). A reverse treatment was also done, where RAW 264.7 cells were pre-incubated with LPS for 1 h and subsequently treated with **1**–**6**. The anti-inflammatory activity of **1**–**6** was unaffected by the treatment regimen. The IC_50_ of **1**–**6** obtained using the two treatment regimens did not show any significant difference (Table 1).

While xestoquinone (**1**) showed comparable activity to *t*BHQ, significant cytotoxicity was observed in **1**. There was no significant difference between the potency of anti-inflammatory activity and cytotoxic effects of xestoquinone (**1**) (Table 1). The cytotoxicity counter-screen also showed that **2** and **3** have direct cytotoxic effects on LPS-stimulated RAW 264.7 cells (IC_50_ = 5.3–6.4 μM) that overlapped with the anti-inflammatory activity. In contrast, **4**–**6** had weak cytotoxicity, with IC_50_ ≥ 16 μM. Compounds **5** and **6** had significantly reduced cytotoxicity compared to xestoquinone (**1**) (Table 1). The decrease in NO production with hydroxymethyl- and methoxy-substituted xestoquinones (**4**–**6**) treatments did not coincide with lower cell viability (Table 1). Notably, -CH_2_OH substitution at C-14 reduced the cytotoxicity of **4** by more than two-fold, compared to **1**. Substitution of the same moiety at C-15 further reduced the cytotoxic effects of **5** by five-fold, compared to **1**. A 3.7-fold decrease in cytotoxicity relative to **1** was observed with -OCH_3_ substitution in **6**. 

### 3.4. Direct Antioxidant Activity of **1**–**6**

**Figure 3:**
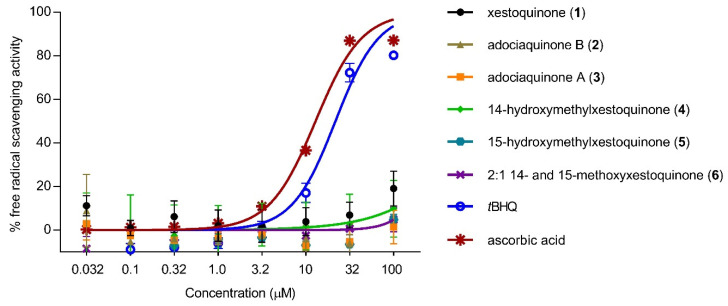
Direct antioxidant effects of xestoquinone and analogues (**1**–**6**). Xestoquinone and derivatives (**1**–**6**) did not show free-radical scavenging activity using the DPPH reagent. *t*BHQ and ascorbic acid served as positive controls and showed a concentration-dependent scavenging activity. Data are presented as mean ± SD, *n* = 3.

To determine whether the decrease in NO production was due to the direct antioxidant effects of **1**–**6**, free radical scavenging activity was evaluated using the DPPH scavenging assay. None of the compounds exhibited free radical scavenging activity up to 100 μM (Figure 3). The positive controls, *t*BHQ and ascorbic acid, showed a concentration-dependent response and exhibited moderate scavenging activity with IC_50_ values of 11 and 13 μM, respectively.

### 3.5. Nrf2 Activation in Nrf2-Luciferase Reporter MCF7 Cells

Due to the conserved Michael acceptor motif in the purified quinone compounds, typical for Nrf2 activators [24,25,26], we looked at the effects of **1**–**6** on the Nrf2-ARE signaling pathway as a potential mechanism for the anti-inflammatory activity. Nrf2 is expressed in all cell types and is usually present at basal cellular levels [27,28]. Hence, Nrf2-luciferase reporter MCF7 cells were used instead of murine macrophage cells to evaluate the ability of the compounds to activate the Nrf2-ARE signaling pathway. 

**Figure 4:**
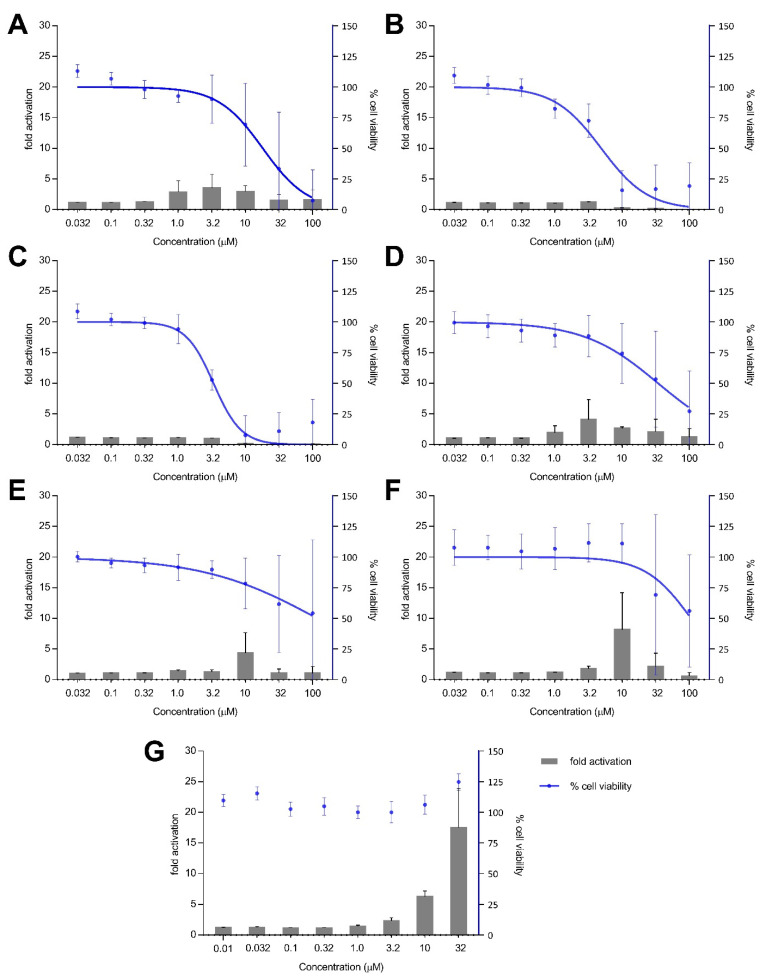
Nrf2-ARE fold activation (bar graph) and cell viability (line graph) effects of (**A**) xestoquinone (**1**), (**B**) adociaquinone B (**2**), (**C**) adociaquinone A (**3**), (**D**) 14-hydroxymethylxestoquinone (**4**), (**E**) 15-hydroxymethylxestoquinone (**5**), (**F**) 2:1 14-methoxyxestoquinone and 15-methoxyxestoquinone (**6**), and (**G**) *t*BHQ on Nrf2-luciferase reporter MCF7 stable cells after 12 h incubation. A modest increase in Nrf2 activation was observed with **1**, **4,** and **6** at 12 h posttreatment. Fold activation is presented as mean + SD, while the % cell viability is presented as mean ± SD from two independent experiments with three replicates each.

**Figure 5:**
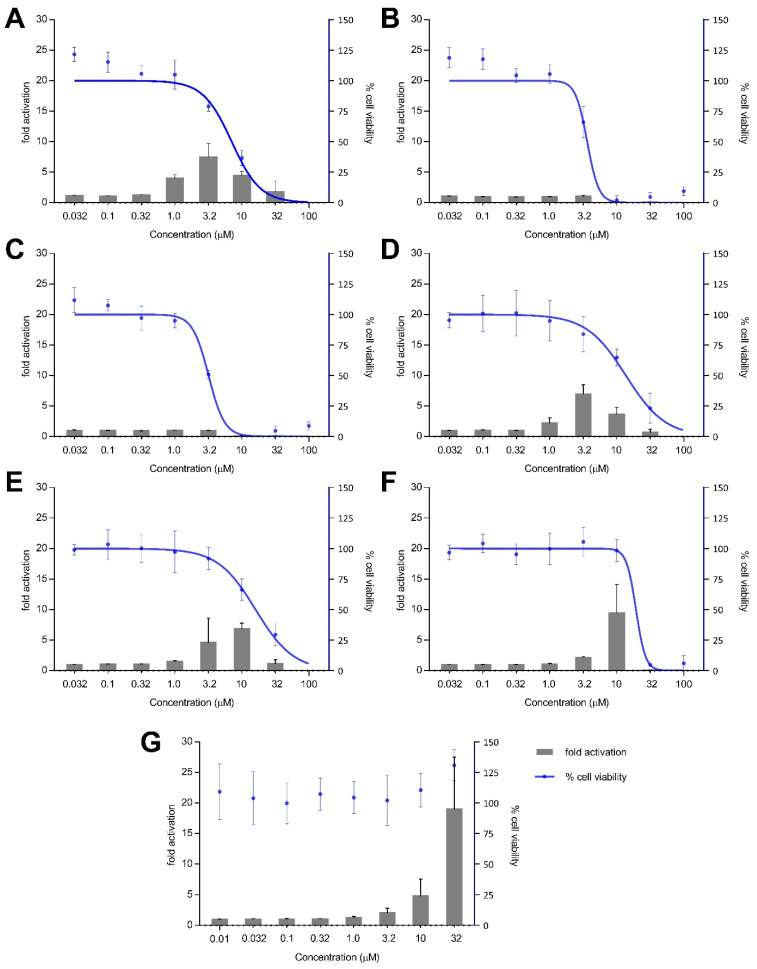
Nrf2-ARE fold activation (bar graph) and cell viability (line graph) effects of (**A**) xestoquinone (**1**), (**B**) adociaquinone B (**2**), (**C**) adociaquinone A (**3**), (**D**) 14-hydroxymethylxestoquinone (**4**), (**E**) 15-hydroxymethylxestoquinone (**5**), (**F**) 2:1 14-methoxyxestoquinone and 15-methoxyxestoquinone (**6**), and (**G**) *t*BHQ on Nrf2-luciferase reporter MCF7 stable cells after 16 h incubation. Xestoquinone (**1**) and the monosubstituted analogues (**4**–**6**) showed significant activation of Nrf2 activity in a reporter assay. The highest Nrf2 activation was observed in **4** and **6**, comparable with the activity of *t*BHQ. Fold activation is presented as mean + SD, while the % cell viability is presented as mean ± SD from two independent experiments with three replicates each.

Treatment with xestoquinone (**1**) for 12 h exhibited at least three-fold Nrf2 activation at 1.0 and 3.2 μM, with no cytotoxicity to Nrf2-MCF7 cells (Figure 4). At 16-h treatment, **1** showed a four- and seven-fold increase in Nrf2 activation at 1.0 and 3.2 μM, respectively, with no significant cytotoxic effects (Figure 5). However, a decrease in Nrf2 activation was observed at higher concentrations, due to the significant cytotoxicity of xestoquinone (**1**). SAR indicated that modifications to the quinone moiety influenced the Nrf2 activation capacity (Figure 5), as observed with the higher concentration required for **4**–**6** at 12-h and 16-h incubation. The highest Nrf2 activation for 14-hydroxymethyl- (**4**) and 15-hydroxymethyl- (**5**) xestoquinones were at seven-fold at 3.2 μM and 10 μM, respectively (16-h treatment period, Figure 5). The 2:1 mixture of 14-methoxyxestoquinone and 15-methoxyxestoquinone (10 μM, **6**) activated Nrf2 by eight- and nine-fold at 12 h and 16 h, respectively (Figure 4 and Figure 5), with no observable cytotoxicity. Nrf2 activation was not observed with adociaquinones B and A (**2** and **3**) at both timepoints. Compounds **1**–**6** did not exhibit significant Nrf2 activation at 8 h posttreatment (Supplementary Figure S34). The known Nrf2 activator, *t*BHQ, demonstrated six- and 18-fold activation at 10 and 32 μM, respectively, with no observable cytotoxicity against Nrf2-MCF7 cells after 12-h (Figure 4). *t*BHQ also displayed non-cytotoxic Nrf2 activation at 16-h incubation with four- and 19-fold activation at 10 and 32 μM, respectively (Figure 5). The maximum Nrf2 stimulation displayed by **4, 5,** and **6** at 10 μM was comparable with the activity of *t*BHQ.

### 3.6. Differential Expression of Pro-Inflammatory (Nos2 and Il1b) and Cytoprotective Antioxidant (Nqo1) Genes

To further determine the cellular basis for the anti-inflammatory activity of **1**–**6**, changes in the transcript level of pro-inflammatory *Nos2* and *Il1b*, and cytoprotective *Nqo1* genes were assessed using quantitative real-time polymerase chain reaction (qRT-PCR). Mouse *Gapdh* was used as the reference gene. Compounds **1**, **2**, **5**, and **6** were used as model compounds since these have different levels of anti-inflammatory activity and Nrf2 activation.

**Figure 6:**
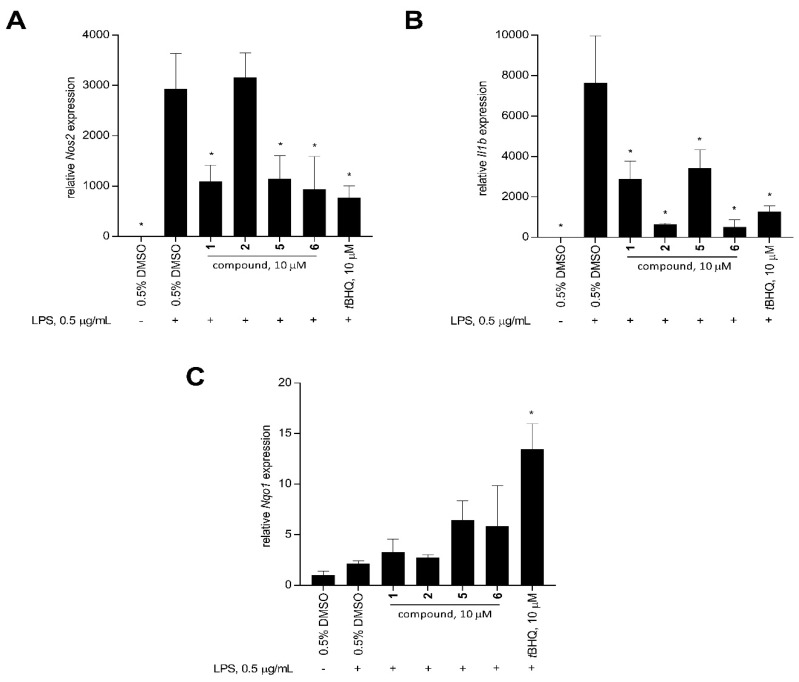
Relative expression (mean + SD, *n* = 3) of pro-inflammatory and cytoprotective genes: (**A**) *Nos2*, (**B**) *Il1b*, and (**C**) *Nqo1* in LPS-stimulated RAW 264.7 murine macrophage cells after 12-h treatment with 10 μM of xestoquinone (**1**), adociaquinone B (**2**), 15-hydroxymethylxestoquinone (**5**), 2:1 mixture of 14- and 15-methoxyxestoquione (**6**), and *t*BHQ (positive control). Mouse *Gapdh* was used as the reference gene. Compound **6** showed comparable activity to *t*BHQ in downregulating *Nos2* and *Il1b* expression. Data are presented as relative gene expression (mean + SD, *n* = 3) of a representative trial. Asterisk (*) denotes significant difference relative to 0.5% DMSO + LPS. Data were analyzed using one-way ANOVA and Tukey’s post-hoc test at *p*-value < 0.05.

Stimulation of RAW 264.7 macrophage cells with LPS significantly increased the expression of *Il1b* and *Nos2* compared to the unstimulated solvent control after 12 h (Figure 6). At 10 μM, xestoquinone (**1**) and 15-hydroxymethylxestoquinone (**5**) significantly downregulated the LPS-induced expression of *Nos2* in macrophage cells by 2.7- and 2.6-fold, respectively. A three-fold decrease in *Nos2* expression was observed with the 2:1 mixture of 14-methoxyxestoquinone and 15-methoxyxestoquinone (**6**, 10 μM), comparable with the positive control, *t*BHQ (3.8-fold, 10 μM, Figure 6). On the other hand, *Nos2* expression in LPS-stimulated cells was unaffected by adociaquinone B (**2**) at 10 μM. Relative to LPS-stimulated solvent control, **1** and **5** downregulated *Il1b* expression by 2.6- and 2.1-fold, respectively, after 12 h. A much larger reduction in *Il1b* expression was observed with **2** and **6**, decreasing by 12.1- and 14.8-fold, respectively. In comparison, *t*BHQ caused a six-fold decrease compared to the LPS-induced *Il1b* gene expression (Figure 6). 

LPS stimulation on macrophage cells had no effect on the *Nqo1* expression (Figure 6). In contrast, *t*BHQ caused a 6.3-fold *Nqo1* upregulation in LPS-stimulated RAW 264.7 at 10 μM (Figure 6). Xestoquinone (**1**) and adociaquinone B (**2**) treatments did not change the *Nqo1* expression, compared to the unstimulated and LPS-stimulated controls. An increase in *Nqo1* expression was observed with treatments of 15-hydroxymethylxestoquinone (**5**) and 2:1 mixture of 14- and 15-methoxyxestoquione (**6**), causing a 3.0- and 2.7-fold upregulation in *Nqo1* expression at 10 μM (Figure 6), respectively. 

## 4. Discussion

In this study, we capitalized on the chemical diversity from marine sponges to discover anti-inflammatory compounds. The anti-inflammatory activity was evaluated in LPS-stimulated murine macrophage cells and an antiproliferative activity testing was also used as a counter-screen. This allowed for selecting priority extracts that decrease NO production in RAW 264.7 cells with minimal effects on the cell viability, hence, excluding promiscuous and non-selective agents. Screening and subsequent validation of the bioactivity identified a *Neopetrosia compacta* sponge as bioactive and the priority for purification. Xestoquinone (**1**), and five analogues, namely adociaquinone B (**2**), adociaquinone A (**3**), 14-hydroxymethylxestoquinone (**4**), 15-hydroxymethylxestoquinone (**5**), and an inseparable 2:1 mixture of 14-methoxyxestoquinone and 15-methoxyxestoquinone (**6**) were purified from *N. compacta* using a bioactivity-guided approach. Xestoquinone and analogues were previously isolated from various marine sponges of the genera *Xestospongia*, *Adocia*, and *Petrosia*. These quinone compounds are known to possess a wide range of biological activities, including anti-proliferative, cardiotonic, and inhibitory activity against regulatory enzymes [18,22,29,30,31,32]. Here, we report the anti-inflammatory activity of these quinoid compounds. Xestoquinone and analogues (**1**–**6**) attenuated the NO production in a concentration-dependent manner in LPS-stimulated RAW 264.7 murine macrophage cells. SAR indicates that the presence of a monosubstituted quinone moiety, as in **4**–**6**, is favorable and modulates the cytotoxic effects, and widens the therapeutic window of xestoquinone analogues (Table 1).

Xestoquinone (**1**) and monosubstituted derivatives **4**–**6** share a common structural feature, a sterically unhindered α,β-unsaturated carbonyl moiety, suggesting that these compounds may exert their anti-inflammatory activity via the activation of the nuclear factor erythroid 2-related factor 2 (Nrf2)through the Keap1/Nrf2-ARE signaling pathway [24,33,34,35]. Most Nrf2 activators possess an electrophilic Michael acceptor motif [24,25,26] that serves as the reactive center in the reaction of the nucleophilic sulfhydryl group of the cysteine residues in Keap1. Covalent modification of the cysteine residues of Keap1 leads to adduct formation and promotes the dissociation of Nrf2 from Keap1 [16,24,25,26,27,36].

Electrophilicity and preferential reactivity of xestoquinone (**1**) were previously demonstrated with the addition of β-mercaptoethanol to yield a disubstituted 2-mercaptoethanol adduct, with C-14 and C-15 as the main reactive centers [37,38]. The xestoquinone analogue halenaquinone, differing from **1** by the lack of C-3 oxidation, also showed preferential reactivity towards thiol nucleophiles. The C-14 and C-15 of halenaquinone served as the electrophilic centers when reacted with N-acetyl-L-cysteine (NAC) to yield the C-14 monosubstituted and C-14-,C-15-disubstituted NAC adducts [39]. We similarly show that xestoquinone (**1**) forms a monosubstituted adduct with NAC (Figure S37). Further, xestoquinone (**1**) modifies specific sulfhydryl groups in the thiol residues of the skeletal muscle myosin [37,38,40]. This conformational change of the myosin consequently led to activation of the actomyosin ATPase, inhibition of Ca^2+^ ATPase activity, and stimulation of Ca^2+^ release from the skeletal muscles [37,38,40]. These reactions serve as a proof-of-concept of the potential adduct formation between xestoquinone (**1**) and Keap1 or other thiol-containing molecules [38,39]. 

Monosubstitution at either C-14 or C-15, as in **4**–**6**, introduced steric hindrance and attenuated the electrophilicity of the quinone moiety. The hydroxymethyl- and methoxy-substitution in the quinone moiety can potentially affect the interaction with Keap1 and, consequently, Nrf2 activation. No Nrf2 activation with the disubstituted derivatives **2** and **3** is expected, since these compounds possess an aromatic sulfone moiety which constrains the interaction with the cysteine residues of Keap1. While the monosubstitution of **4**–**6** led to a slight decrease in the potency of Nrf2 activation, this was ideal in reducing the cytotoxic effects of these compounds, thereby increasing the selectivity index. 

The reaction of Michael acceptors with Nrf2 results in dissociation from Keap1, stabilization of Nrf2, and subsequent translocation to the nucleus [24,25,26]. This then triggers the binding of Nrf2 to the antioxidant-responsive element (ARE) promoter region and, consequently, gene expression of phase II detoxifying and cytoprotective enzymes such as NADPH quinone oxidoreductase I (NQO1), glutathione S-transferase (GST), and heme oxygenase-1 (HO-1) [16,24,26,27,36]. Upregulation of these Nrf2-target genes leads to a decreased expression of inflammatory mediators downstream of the pathway. The expression of inflammatory mediators and effector molecules such as TNF-α, IL-1β, IL-6, COX-2, and iNOS at transcript and protein levels in various mammalian cells is attenuated upon Nrf2 activation [16,24,26,27,28,34]. Differential gene expression demonstrated that xestoquinone and monosubstituted derivatives downregulated the pro-inflammatory genes *Nos2* and *Il1b*, leading to a downstream reduction in NO production (Figure 6). Compound **6** caused the highest attenuation in *Nos2* and *Il1b* expression and an increase in *Nqo1* transcript level.

Adociaquinones, xestoquinone, and hydroxymethylated analogues are also known inhibitors of HIF-1 activation in hypoxia- and chemical-induced reporter assay [22]. While adociaquinones showed higher attenuation of HIF-1 in the reporter assay, 14-hydroxymethylxestoquinone showed more potent activity in blocking VEGF production in both low oxygen (1% O_2_) and chemical-induced hypoxia [22]. The HIF-1 inhibitory activity of 14-hydroxymethylxestoquinone was comparable to the positive control cycloheximide. HIF-1 is important in inflammation, and its expression is induced by LPS in various cell types [41,42,43,44]. In vivo administration of LPS in BALB/c mice showed a concomitant increase in HIF-1, TNFα, IL-6, and KC transcript levels in lung epithelial cells [43]. Intraperitoneal administration of propofol (50 mg/kg) attenuated HIF-1α expression, in parallel with the decrease in TNFα, IL-6, and KC expression [43]. Further, HIF-1 and NO also have multiple interactions, with cellular responses to both HIF-1 and NO being intricately regulated by one another [41,42,45]. HIF-1 is a master regulator of inflammation and increases NO production through upregulation of NOS2 and COX4-2 expression [45]. In BALB/c-OVA model of asthma, the HIF-1α antagonist YC-1 suppressed HIF-1α expression and lowered the transcript levels of IL-5, IL-13, myeloperoxidase, and NOS2 [46]. 

The anti-inflammatory activity of monosubstituted xestoquinones may then be mediated by the dual effects on modulation of HIF-1 and Nrf2 activities. The less studied **4**–**6** offer the advantage of lower toxicity and potent anti-inflammatory activity. Minor modifications in the quinoid moiety allow for the fine-tuning of the bioactivity of the xestoquinone family of compounds. 

## 5. Conclusions

We demonstrate the potential of xestoquinone and monosubstituted derivatives as anti-inflammatory agents and Nrf2 activators. While the anti-inflammatory activity was unaffected by structural modifications in the para-quinone moiety, the monosubstituted analogues had significantly reduced cytotoxicity. The partial functionalization of the para-quinone moiety significantly improved the selectivity of the monosubstituted analogues of xestoquinone. The anti-inflammatory activity and Nrf2 activation by xestoquinone and monosubstituted derivatives were correlated to the downregulation of the pro-inflammatory *Nos2* and *Il1b* genes, leading to a decrease in NO production.

## Figures and Tables

**Table 1:** Anti-inflammatory activity (IC_50,_ μM) and cytotoxicity (IC_50,_ μM) of **1**–**6** in LPS-stimulated RAW 264.7 murine macrophage cells (ATCC^®^TIB-71™) after pre-treatment with compound for 1 h followed by the addition of LPS, and pre-treatment with LPS for 1 h followed by the addition of compound.

Compound	Compound Pre-Treatment + LPS *^a^*		LPS Pre-Treatment + Compound *^b^*	
	NO Production (μM)	Cell Viability (μM)	NO Production (μM)	Cell Viability (μM)
xestoquinone (**1**)	1.2 ± 0.10	6.0 ± 3.2	0.99	4.9
adociaquinone B (**2**)	1.9 ± 0.72	5.3 ± 2.0	1.5	4.2
adociaquinone A (**3**)	2.1 ± 0.17 *^c^*	6.4 ± 1.2	1.5	5.9
14-hydroxymethylxestoquinone (**4**)	2.5 ± 0.39 *^c^*	16 ± 0.68	1.8	27
15-hydroxymethylxestoquinone (**5**)	4.0 ± 2.4 *^c^*	>32 *^d^*	4.7	>32
2:1 mixture of 14-methoxyxestoquinone and 15-methoxyxestoquinone (**6**)	1.4 ± 0.56 *^c^*	22 ± 8.5 *^d^*	1.6	17
*t*BHQ	1.2 ± 0.23	>100	0.42	>100
dexamethasone	>0.032	>100	>0.032	>100

*^a^* Data are presented as mean ± SD of two independent experiments performed in triplicate. *^b^* Data are presented as mean from a single independent experiment performed in triplicate. *^c^* Indicates significant differences between the IC_50_ of NO production and cytotoxicity of the compound. Data were analyzed using one-tailed unpaired *t*-test at *p*-value < 0.05. *^d^* Denotes significant difference of the cell viability IC_50_ relative to xestoquinone (**1**). Data were analyzed using one-way ANOVA and Tukey’s post-hoc test at *p*-value < 0.05.

## Data Availability

The data are contained within the article and supplementary materials.

## Supplementary Materials

The following supporting information can be downloaded at: https://www.mdpi.com/article/10.3390/antiox11040607/s1, Table S1: Anti-inflammatory activity of sponge crude extracts; Figure S1: Screening for anti-inflammatory activity of sponge crude extracts at 5 μg/mL; Figures S2–S31; UV profile, HRESIMS spectrum, 1D- and 2D-NMR spectra of compounds **1**–**6**; Figures S32–S34: Dose-response curves of the anti-inflammatory activity of compounds **1**–**6**; Figure S35: Nrf2 activation and cell viability of Nrf2-luciferase reporter MCF7 stable cells after 8 h incubation with compounds **1**–**6**; Figure S36: Relative gene expression of *Nos2*, *Il1b*, and *Nqo1* after treatment with **1**, **2**, and **6**; Figure S37: HRESIMS spectrum for xestoquinone (**1**) and NAC Michael addition reaction.
