# Supplementary material for: Anti-Inflammatory Activity of Monosubstituted Xestoquinone Analogues from the Marine Sponge Neopetrosia compacta
Source: Antioxidants (Basel). 2022 Mar 22;11(4):607. doi: 10.3390/antiox11040607 (PMC9028180; doi:10.3390/antiox11040607)
Supplement: Supplementary file 1 [file antioxidants-11-00607-s001.zip › antioxidants-1591201-supplementary.pdf]

Supplementary Information

Anti-inflammatory Activity of Monosubstituted Xestoquinone Analogues from the Marine Sponge *Neopetrosia compacta*

Shalice R. Susana and **Lilibeth A. Salvador-Reyes***

Marine Science Institute, University of the Philippines – Diliman, Velasquez St., UP Diliman, Quezon City, 1101 Philippines

*lsreyes@msi.upd.edu.ph

Contents:

Table S1. Mean % NO production and % cell viability of LPS-stimulated RAW 264.7 cells after treatment with hexane (h), (4:1) CH_2Cl_2:MeOH (d), and MeOH (m) crude extracts 5

Figure S1. Screening for anti-inflammatory activity of 231 marine sponge crude extracts based on attenuation of (**A**) NO production and (**B**) cell viability counterscreen at 5 µg/mL in LPS-stimulated RAW 264.7 cells. Each dot represents the %NO production (**A**) or %cell viability (**B**) of LPS-stimulated RAW 264.7 cells in response to the 5 µg/mL extract treatment. Lipophilic extracts are shown in green, while semipolar and polar extracts are displayed in lilac and brown, respectively. The (4:1) CH_2Cl_2: MeOH extract of *N. compacta* Lala1F sponge, highlighted in yellow, reduced the NO production by 51% at 5 µg/mL, respectively, with no observable cytotoxic effects. The positive controls dexamethasone (50 µM) and *t*BHQ (10 µM) reduced NO production by 50% and 90%, respectively, with no observed cytotoxicity. Data presented as mean of %NO production or %cell viability (n=3). 11

Figure S2. UV profile of xestoquinone (**1**) in CH_3CN 14

Figure S3. HRESIMS spectrum of xestoquinone (**1**) in positive mode 15

Figure S4. ^{1}H NMR spectrum of xestoquinone (**1**) in $CDCl_3$ (500 MHz) 16

Figure S5. COSY spectrum of xestoquinone (**1**) in $CDCl_3$ (500 MHz) 17

Figure S6. HSQC spectrum of xestoquinone (**1**) in $CDCl_3$ (500 MHz) 18

Figure S7. HMBC spectrum of xestoquinone (**1**) in $CDCl_3$ (500 MHz) 19

Figure S8. UV profile of adociaquinone B (**2**) in CH_3CN 20

Figure S9. HRESIMS spectrum of adociaquinone B (**2**) in positive mode 21

Figure S10. ^{1}H NMR spectrum of adociaquinone B (**2**) in DMSO-d_6 (500 MHz) 22

Figure S11. COSY spectrum of adociaquinone B (**2**) in DMSO-d_6 (500 MHz) 23

Figure S12. HSQC spectrum of adociaquinone B (**2**) in DMSO-d_6 (500 MHz) 24

Figure S13. UV profile of adociaquinone A (**3**) in CH_3CN 25

Figure S14. HRESIMS spectrum of adociaquinone A (**3**) in positive mode 26

Figure S15. ^{1}H NMR spectrum of adociaquinone A (**3**) in DMSO-d_6 (500 MHz) 27

Figure S16. COSY spectrum of adociaquinone A (**3**) in DMSO-d_6 (500 MHz) 28

Figure S17. UV profile of 14-hydroxymethylxestoquinone (**4**) in CH_3CN 29

Figure S18. HRESIMS spectrum of 14-hydroxymethylxestoquinone (**4**) in positive mode 30

Figure S19. ^1H NMR spectrum of 14-hydroxymethylxestoquinone (**4**) in $CDCl_3$ (500 MHz) 31

Figure S20. COSY spectrum of 14-hydroxymethylxestoquinone (**4**) in $CDCl_3$ (500 MHz) 32

Figure S21. HSQC spectrum of 14-hydroxymethylxestoquinone (**4**) in $CDCl_3$ (500 MHz) 33

Figure S22. HMBC spectrum of 14-hydroxymethylxestoquinone (**4**) in $CDCl_3$ (500 MHz) 34

Figure S23. UV profile of 15-hydroxymethylxestoquinone (**5**) in CH_3CN 35

Figure S24. HRESIMS spectrum of 15-hydroxymethylxestoquinone (**5**) in positive mode 36

Figure S25. ^1H NMR spectrum of 15-hydroxymethylxestoquinone (**5**) in $CDCl_3$ (500 MHz) 37

Figure S26. COSY spectrum of 15-hydroxymethylxestoquinone (**5**) in $CDCl_3$ (500 MHz) 38

Figure S27. UV profile of 2:1 mixture of 14- and 15-methoxyxestoquinone (**6**) in CH_3CN 39

Figure S28. HRESIMS spectrum of 2:1 mixture of 14- and 15-methoxyxestoquinone (**6**) in positive mode 40

Figure S29. ^1H NMR spectrum of 2:1 mixture of 14- and 15-methoxyxestoquinone (**6**) in $CDCl_3$ (500 MHz) 41

Figure S30. COSY spectrum of 2:1 mixture of 14- and 15-methoxyxestoquinone (**6**) in $CDCl_3$ (500 MHz) 42

Figure S31. HSQC spectrum of 2:1 mixture of 14- and 15-methoxyxestoquinone (**6**) in $CDCl_3$ (500 MHz) 43

Figure S32. NO production and cell viability of LPS-stimulated RAW264.7 macrophage cells (mean ± SD, n = 3) after pre-treatment with **A**. xestoquinone (**1**), **B**. adociaquinone B (**2**), **C**. adociaquinone A (**3**), **D**. 14-hydroxymethylxestoquinone (**4**), **E**. 15-hydroxymethylxestoquinone (**5**), **F**. 2:1 14-methoxyxestoquinone and 15-methoxyxestoquinone (**6**), **G**. *t*BHQ, and **H**. dexamethasone for 1 h followed by the addition of LPS (Experiment 1). 44

Figure S33. NO production and cell viability of LPS-stimulated RAW264.7 macrophage cells (mean ± SD, n = 3) after pre-treatment with **A**. xestoquinone (**1**), **B**. adociaquinone B (**2**), **C**. adociaquinone A (**3**), **D**. 14-hydroxymethylxestoquinone (**4**), **E**. 15-hydroxymethylxestoquinone (**5**), **F**. 2:1 14-methoxyxestoquinone and 15-methoxyxestoquinone (**6**), **G**. *t*BHQ, and **H**. dexamethasone for 1 h followed by the addition of LPS (Experiment 2). 45

Figure S34. NO production and cell viability of LPS-stimulated RAW264.7 macrophage cells (mean ± SD, n = 3) after using the reverse regimen of pre-treatment with LPS for 1h and followed

by the addition of **A**. xestoquinone (**1**), **B**. adociaquinone B (**2**), **C**. adociaquinone A (**3**), **D**. 14-hydroxymethylxestoquinone (**4**), **E**. 15-hydroxymethylxestoquinone (**5**), **F**. 2:1 14-methoxyxestoquinone and 15-methoxyxestoquinone (**6**), **G**. *t*BHQ, and **H**. dexamethasone. (Experiment 1). 46

Figure S35. Nrf2-ARE activation (bar graph, data presented as mean + SD, n = 3) and cell viability (line graph, data presented as mean ± SD, n = 3) effects of **A**. xestoquinone (**1**), **B**. adociaquinone B (**2**), **C**. adociaquinone A (**3**), **D**. 14-hydroxymethylxestoquinone (**4**), **E**. 15-hydroxymethylxestoquinone (**5**), **F**. 2:1 14-methoxyxestoquinone and 15-methoxyxestoquinone (**6**), and **G**. *t*BHQ on Nrf2-luciferase reporter MCF7 stable cells after 8 h incubation. (Experiment 1). 47

Figure S36. Relative expression (mean + SD, n = 3) of pro-inflammatory and cytoprotective genes: (**A**) *Nos2*, (**B**) *Il1b*, and (**C**) *Nqo1* in LPS-stimulated RAW 264.7 murine macrophage cells after 12-h treatment with 10 μM of xestoquinone (**1**), adociaquinone B (**2**), 2:1 mixture of 14- and 15-methoxyxestoquione (**6**), and *t*BHQ (positive control). Compoubd **5** was not tested due to insufficient material. Mouse *Gapdh* was used as reference gene. Compound **6** showed comparable activity to *t*BHQ in downregulating *Nos2* and *Il1b* expression. Asterisk (*) denotes significant difference relative to 0.5% DMSO + LPS. Data analyzed using one-way ANOVA and Tukey's post-hoc test at p-value < 0.05. (Experiment 2) 48

Figure S37. HRESIMS spectra of the reaction of (A) 2:1 and (B) 50:1 *N*-acetyl cysteine (NAC) and xestoquinone (**1**) in positive mode 49

Table S1. Mean % NO production and % cell viability of LPS-stimulated RAW 264.7 cells after treatment with hexane (h), (4:1) CH_2Cl_2:MeOH (d), and MeOH (m) crude extracts

extract	% NO production		% cell viability	
	50 µg/mL	5 µg/mL	50 µg/mL	5 µg/mL
**BL-15-A001.h**	23.60	38.04	9.70	108.60
**BL-15-A001.d**	9.90	17.30	1.60	55.30
**BL-15-A001.m**	92.40	104.30	83.60	100.10
**BL-15-A002.h**	124.70	37.08	2.80	77.20
**BL-15-A002.d**	16.40	45.20	113.50	105.00
**BL-15-A002.m**	94.40	90.95	106.70	110.30
**BL-15-A004.h**	96.26	90.95	74.29	106.64
**BL-15-A004.d**	28.09	10.11	98.40	97.00
**BL-15-A004.m**	101.30	95.68	104.80	102.40
**BML-15-1.h**	13.50	72.81	80.80	118.60
**BML-15-1.d**	99.00	100.00	110.10	103.60
**BML-15-1.m**	59.00	50.90	105.20	106.80
**BML-15-2.h**	16.80	64.04	74.50	94.90
**BML-15-2.d**	109.50	111.60	109.40	104.50
**BML-15-2.m**	56.80	64.50	98.80	104.60
**BML-15-3.h**	1.10	5.62	0.40	48.70
**BML-15-3.d**	40.40	94.50	79.60	105.30
**BML-15-3.m**	80.00	91.27	110.00	114.70
**BML-15-4.h**	3.40	10.11	0.30	74.20
**BML-15-4.d**	6.90	10.63	38.00	68.50
**BML-15-4.m**	110.40	116.85	112.20	116.40
**BML-15-5.h**	15.50	81.03	93.20	104.20
**BML-15-5.d**	66.10	106.05	109.80	121.90
**BML-15-5.m**	3.50	56.20	28.90	94.20
**BML-15-6.h**	21.30	23.60	73.00	87.90
**BML-15-6.d**	44.10	93.52	101.00	110.50
**BML-15-6.m**	94.60	98.70	92.20	102.40
**BML-15-11.h**	-3.40	64.04	87.80	101.80
**BML-15-11.d**	89.10	90.80	86.80	101.80
**BML-15-11.m**	23.40	13.63	95.00	87.40
**BML-15-12.h**	21.90	92.35	110.70	85.50
**BML-15-12.d**	94.40	89.20	103.10	111.00
**BML-15-12.m**	87.50	96.11	105.60	103.70
**BML-15-13.h**	7.90	46.07	86.10	106.40
**BML-15-13.d**	95.20	93.90	99.40	101.10
**BML-15-13.m**	42.20	53.00	106.20	105.90
**BML-15-14.h**	20.20	111.24	81.50	100.60
**BML-15-14.d**	73.70	71.78	97.50	79.50
**BML-15-14.m**	68.40	69.43	87.10	96.50
**BML-15-15.h**	28.10	94.38	9.70	108.60

Table S1. Continuation

extract	% NO production		% cell viability	
	50 μg/mL	5 μg/mL	50 μg/mL	5 μg/mL
**BML-15-15.d**	98.30	93.50	100.00	99.90
**BML-15-15.m**	47.70	50.90	80.30	104.50
**BML-15-16.h**	10.10	41.57	2.80	77.20
**BML-15-16.d**	92.00	56.73	93.90	98.50
**BML-15-16.m**	116.20	103.30	102.10	101.90
**BML-15-17.h**	33.40	92.04	89.40	92.90
**BML-15-17.d**	92.20	92.50	103.00	99.50
**BML-15-17.m**	119.50	91.30	102.10	107.10
**BML-15-18.h**	5.60	53.93	74.30	96.30
**BML-15-18.d**	87.10	89.40	100.30	96.20
**BML-15-18.m**	82.10	101.70	102.70	102.10
**BML-15-19.h**	7.90	41.60	74.50	94.90
**BML-15-19.d**	90.50	92.80	96.30	99.80
**BML-15-19.m**	107.90	106.70	105.40	101.80
**BML-15-20.h**	11.00	44.80	5.10	83.30
**BML-15-20.d**	57.00	69.80	108.70	121.90
**BML-15-20.m**	89.60	97.40	104.20	99.40
**BML-15-21.h**	16.40	94.40	63.50	81.10
**BML-15-21.d**	65.70	79.10	105.20	70.90
**BML-15-21.m**	54.80	19.10	68.10	63.20
**BML-15-22.h**	16.80	80.90	0.40	48.70
**BML-15-22.d**	107.80	109.20	102.10	105.60
**BML-15-22.m**	111.60	102.90	101.40	101.30
**BML-15-23.h**	100.00	70.80	0.30	74.20
**BML-15-23.d**	55.80	7.30	57.90	67.80
**BML-15-23.m**	104.20	109.10	105.80	100.00
**BML-15-24.h**	50.90	124.10	125.10	117.00
**BML-15-24.d**	80.56	84.02	106.90	114.99
**BML-15-24.m**	84.02	92.22	102.12	101.50
**BML-15-25.h**	36.34	97.68	95.46	86.74
**BML-15-25.d**	87.04	97.19	93.92	118.21
**BML-15-25.m**	91.79	100.86	102.03	97.85
**BML-15-26.d**	97.00	101.50	110.85	104.84
**BML-15-26.m**	81.70	100.00	99.92	101.97
**BML-15-27.h**	23.60	80.90	81.49	100.56
**BML-15-27.d**	85.31	88.34	104.92	118.35
**BML-15-27.m**	105.62	103.02	95.85	97.23
**BML-15-28.h**	14.91	102.56	128.33	153.44
**BML-15-28.d**	99.60	101.50	101.80	104.04
**BML-15-28.m**	88.80	97.50	85.52	98.06
**BML-15-30.d**	107.00	97.40	99.49	103.10

Table S1. Continuation

extract	% NO production		% cell viability	
	50 µg/mL	5 µg/mL	50 µg/mL	5 µg/mL
**BML-15-30.m**	107.10	98.80	97.66	97.50
**BML-15-31.h**	32.82	93.28	100.54	99.64
**BML-15-31.d**	61.56	89.63	115.56	116.77
**BML-15-31.m**	106.39	100.00	116.15	126.99
**BML-15-32.h**	13.63	91.84	64.33	104.96
**BML-15-32.d**	107.00	97.40	103.01	103.15
**BML-15-32.m**	104.20	94.20	102.82	97.79
**BML-15-33.h**	16.19	92.00	69.07	95.55
**BML-15-33.d**	89.20	94.60	102.06	105.54
**BML-15-33.m**	116.20	100.40	103.12	96.30
**BML-15-34.h**	50.56	91.01	75.81	93.15
**BML-15-34.d**	81.43	89.63	105.91	111.09
**BML-15-34.m**	94.70	86.15	103.81	119.81
**CG1E.h**	94.15	91.96	81.26	108.71
**CG1E.d**	6.36	27.37	24.58	67.45
**CG1E.m**	17.22	77.42	98.43	100.51
**CG1F.h**	99.51	102.44	98.40	109.51
**CG1F.d**	109.60	93.30	98.70	98.15
**CG1F.m**	124.81	95.23	92.42	85.57
**CG1G.h**	-2.10	90.36	103.68	119.17
**CG1G.d**	8.86	82.44	85.15	101.04
**CG1G.m**	75.15	77.19	101.88	110.49
**CG1H.h**	-1.23	97.37	6.18	113.34
**CG1H.d**	116.60	94.50	104.28	103.87
**CG1H.m**	101.22	80.45	92.02	111.26
**CG1J.h**	18.93	98.69	120.16	93.14
**CG1J.d**	66.35	79.52	91.90	103.83
**CG1J.m**	73.52	60.90	93.41	103.29
**CG1K.h**	21.56	114.46	81.43	84.21
**CG1K.d**	74.50	101.50	107.65	102.41
**CG1K.m**	35.60	100.00	108.47	103.27
**CG1L.h**	8.41	97.81	57.77	70.95
**CG1L.d**	70.32	83.28	95.54	104.91
**CG1L.m**	70.26	58.04	86.05	96.92
**CG2E.h**	1.84	90.36	69.77	80.47
**CG2E.d**	76.59	71.99	108.78	102.84
**CG2E.m**	77.19	60.08	87.19	93.55
**CG2F.h**	5.46	97.81	85.19	80.22
**CG2F.m**	61.23	61.13	91.94	104.01
**CG2F.d**	49.83	77.42	108.10	94.01
**CG2J.h**	10.33	16.18	39.22	63.15

Table S1. Continuation

extract	% NO production		% cell viability	
	50 µg/mL	5 µg/mL	50 µg/mL	5 µg/mL
**CG2J.d**	89.60	98.90	101.84	100.60
**CG2J.m**	20.90	136.30	49.14	107.21
**CG2H.h**	3.51	79.04	68.95	83.00
**CG2H.d**	98.90	97.00	89.52	102.53
**CG2H.m**	63.38	87.04	106.36	96.01
**CG2I.h**	82.94	85.87	111.12	113.88
**CG2I.d**	70.30	72.71	109.33	111.53
**CG2I.m**	115.27	108.27	97.22	87.82
**CG2L.h**	-0.39	65.40	77.40	82.27
**CG2L.d**	60.00	50.70	101.66	99.06
**CG2L.m**	84.79	71.83	84.07	93.55
**CG2M.d**	83.35	81.10	100.61	90.12
**CG2M.m**	73.93	75.56	96.27	109.03
**CG3D.h**	2.76	44.78	112.43	102.16
**CG3D.d**	46.40	49.40	98.46	100.32
**CG3D.m**	57.18	79.72	96.84	95.62
**CG3E.h**	12.36	-3.24	3.79	74.88
**CG3E.d**	84.25	85.15	94.30	87.24
**CG3E.m**	78.59	90.42	110.44	105.85
**CG3F.h**	1.56	47.18	0.61	51.63
**CG3F.d**	45.20	49.20	90.29	98.61
**CG3F.m**	86.40	114.60	84.02	102.97
**CG3G.h**	82.46	76.61	108.53	114.71
**CG3G.d**	147.07	126.08	99.12	99.42
**CG3G.m**	98.09	93.32	97.47	92.49
**CG3H.h**	1.56	39.98	81.65	99.13
**CG3H.d**	19.89	79.75	83.06	89.01
**CG3H.m**	79.23	78.82	103.14	105.95
**Lala1C.h**	57.98	54.38	94.74	102.08
**Lala1C.d**	95.05	75.25	98.72	98.82
**Lala1C.m**	113.24	101.13	89.36	111.22
**Lala1D.h**	9.99	86.95	48.39	88.66
**Lala1D.d**	3.98	43.74	45.16	103.86
**Lala1D.m**	2.54	38.03	17.26	96.51
**Lala1E.h**	71.21	93.28	72.41	98.23
**Lala1E.d**	55.58	83.19	42.89	85.86
**Lala1E.m**	17.22	73.66	23.64	39.12
**Lala1F.h**	9.29	23.75	1.02	111.92
**Lala1F.d**	24.10	48.80	99.75	102.66
**Lala1F.m**	55.65	78.07	84.11	80.08
**Lala1G.h**	30.11	113.58	71.07	106.13

Table S1. Continuation

extract	% NO production		% cell viability	
	50 µg/mL	5 µg/mL	50 µg/mL	5 µg/mL
**Lala1G.d**	9.54	92.35	42.41	92.44
**Lala1G.m**	3.96	26.77	18.25	84.06
**Lala1H.h**	101.75	133.74	97.93	96.59
**Lala1H.d**	60.70	56.60	100.97	96.40
**Lala1H.m**	62.10	150.90	87.87	105.19
**Lala1I.h**	-0.35	9.73	0.47	54.79
**Lala1I.d**	3.30	50.10	4.79	99.35
**Lala1I.m**	95.20	68.79	104.85	99.34
**Lala2E.h**	14.54	50.41	1.63	122.07
**Lala2E.d**	18.69	88.64	124.12	100.81
**Lala2E.m**	12.42	56.01	107.75	114.60
**Lala2F.h**	2.76	47.18	17.05	91.07
**Lala2F.d**	24.69	68.04	26.42	87.34
**Lala2F.m**	62.53	70.67	80.92	114.51
**Lala2G.h**	80.55	88.84	94.97	103.55
**Lala2G.d**	71.92	85.75	108.63	98.50
**Lala2G.m**	80.35	83.16	94.35	97.71
**Lala2H.h**	72.42	92.03	112.37	96.80
**Lala2H.d**	47.73	89.20	116.00	98.64
**Lala2H.m**	66.31	83.47	104.99	97.85
**Lala2I.h**	45.98	72.39	105.01	108.08
**Lala2I.d**	66.20	66.94	87.24	88.86
**Lala2I.m**	97.60	75.99	102.08	97.21
**Lala2J.h**	14.77	106.60	71.52	91.70
**Lala2J.d**	66.25	83.80	103.28	86.13
**Lala2J.m**	54.38	67.01	93.84	116.21
**Lala3C.h**	1.40	98.25	51.48	103.37
**Lala3C.d**	107.53	102.51	127.59	128.89
**Lala3C.m**	70.99	66.76	86.74	90.32
**Lala3E.h**	7.54	11.04	0.65	84.61
**Lala3E.d**	66.40	54.50	107.50	108.16
**Lala3E.m**	48.17	61.69	92.55	89.80
**Lala3F.h**	1.40	102.63	43.66	109.50
**Lala3F.d**	74.08	98.75	111.30	109.41
**Lala3F.m**	88.73	60.85	82.61	90.88
**M1C.h**	15.16	41.67	9.08	82.96
**M1C.d**	58.20	105.90	94.30	99.01
**M1C.m**	28.72	73.52	78.66	114.89
**M1D.h**	55.36	99.68	128.00	109.91
**M1D.d**	82.29	114.25	123.50	120.39
**M1D.m**	82.84	85.96	109.80	107.38

Table S1. Continuation

extract	% NO production		% cell viability	
	50 µg/mL	5 µg/mL	50 µg/mL	5 µg/mL
**M1E.h**	13.58	87.72	142.37	113.46
**M1E.d**	20.77	78.39	95.12	90.45
**M1E.m**	70.68	88.15	111.89	106.64
**M1G.h**	83.90	102.55	139.18	135.51
**M1G.d**	72.79	94.38	87.99	111.01
**M1G.m**	78.48	83.47	107.47	96.60
**M1H.h**	42.28	88.20	113.47	113.26
**M1H.d**	61.56	91.36	105.37	110.47
**M1H.m**	88.46	87.21	90.05	99.57
**M1I.h**	94.26	96.49	99.89	76.09
**M1I.d**	52.58	69.99	89.39	96.01
**M1I.m**	49.67	48.26	87.18	96.12
**M1J.h**	97.13	93.46	93.60	93.92
**M1J.d**	74.51	88.34	102.47	112.33
**M1J.m**	95.95	91.27	112.13	111.10
**M1L.h**	78.16	101.12	102.77	96.16
**M1L.d**	57.67	83.15	104.80	107.84
**M1L.m**	95.20	68.79	104.85	99.34
**M1M.h**	18.05	84.06	121.74	100.50
**M1M.d**	18.36	83.59	93.59	108.45
**M1M.m**	92.20	94.70	112.92	111.50
**M3E.h**	94.64	76.61	100.37	110.93
**M3E.d**	87.33	81.97	105.41	110.91
**M3E.m**	100.95	101.91	79.44	87.17
**M3F.h**	92.20	98.54	123.88	122.92
**M3F.d**	71.73	78.07	105.51	104.96
**M3F.m**	102.54	121.95	101.15	87.20

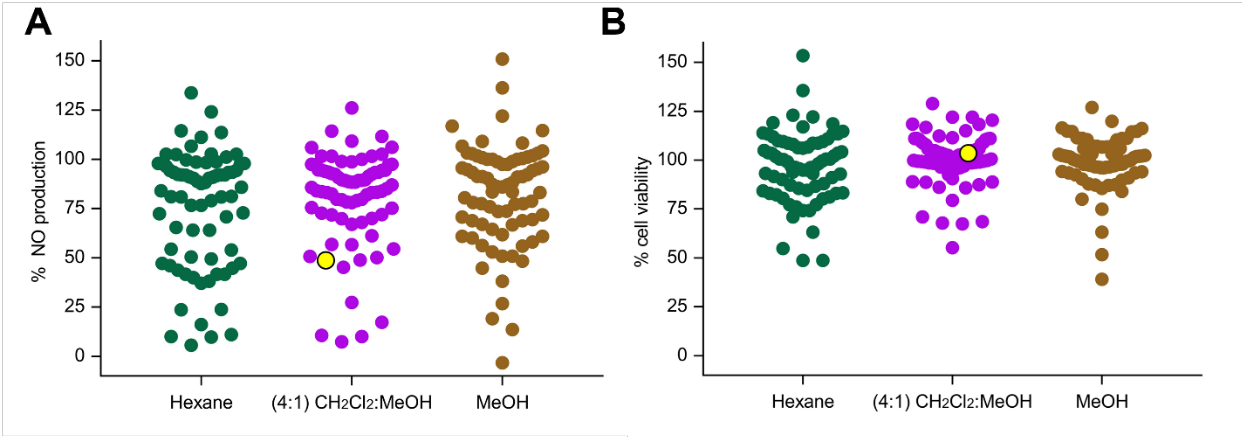

Figure S1. Screening for anti-inflammatory activity of 231 marine sponge crude extracts based on attenuation of (**A**) NO production and (**B**) cell viability counterscreen at 5 µg/mL in LPS-stimulated RAW 264.7 cells. Each dot represents the %NO production (**A**) or %cell viability (**B**) of LPS-stimulated RAW 264.7 cells in response to the 5 µg/mL extract treatment. Lipophilic extracts are shown in green, while semipolar and polar extracts are displayed in lilac and brown, respectively. The (4:1) CH_2Cl_2: MeOH extract of *N. compacta* Lala1F sponge, highlighted in yellow, reduced the NO production by 51% at 5 µg/mL, respectively, with no observable cytotoxic effects. The positive controls dexamethasone (50 µM) and *t*BHQ (10 µM) reduced NO production by 50% and 90%, respectively, with no observed cytotoxicity. Data presented as mean %NO production or %cell viability (n=3).

Structure Elucidation of Compounds

Xestoquinone (**1**): light yellow solid; UV (CH_3CN) λ_{max} 218, 254, 299 nm; LRESIMS *m/z* 319 $[M+H]^+$; HRESIMS *m/z* 319.1204 $[M+H]^+$ (calcd for $C_{20}H_{15}O_4$, 319.0970); ^1H NMR ($CDCl_3$, 500 MHz) δ_H 7.54 (1H, s, H-1), 2.87 (1H, m, H-3a), 2.66 (1H, m, H-3b), 2.26 (1H, m, H-4a), 2.18 (1H, m, H-4b), 2.57 (1H, dt, *J* = 12.9, 3.6 Hz, H-5a), 1.76 (1H, td, *J* = 13.1, 4.4 Hz, H-5b), 9.05 (1H, d, *J* = 10.3 Hz, H-11), 7.04 (1H, d, *J* = 10.3 Hz, H-14), 7.06 (1H, s, H-15), 8.25 (1H, s, H-18), 1.53 (3H, s, H-20); ^{13}C NMR ($CDCl_3$, 125 MHz) δ_C 144.9 (CH, C-1), 121.4 (C, C-2), 16.8 (CH_2, C-3), 18.3 (CH_2, C-4), 31.1 (CH_2, C-5), 37.2 (C, C-6), 147.1 (C, C-7), 144.0 (C, C-8), 170.2 (C, C-9), 137.9 (C, C-10), 127.0 (CH, C-11), 133.2 (C, C-12), 183.7 (C, C-13), 138.6 (CH, C-14), 139.3 (CH, C-15), 184.6 (C, C-16), 130.3 (C, C-17), 123.1 (CH, C-18), 156.1 (C, C-19), 32.4 (CH_3, C-20); MS, ^1H NMR and ^{13}C NMR data are identical with literature values [18,19].

Adociaquinone B (**2**): yellow solid; UV (CH_3CN) λ_{max} 223, 278, 295 nm; LRESIMS *m/z* 424 $[M+H]^+$; HRESIMS *m/z* 424.0862 $[M+H]^+$ (calcd for $C_{22}H_{18}NO_6S$, 424.0855); ^1H NMR (DMSO-*d*$_6$, 500 MHz) δ_H 7.96 (1H, s, H-1), 2.83 (1H, dd, *J* = 16.9, 7.8 Hz, H-3a), 2.56 (1H, m, H-3b), 2.20 (1H, m, H-4a), 2.04 (1H, m, H-4b), 2.60 (1H, m, H-5a), 1.62 (1H, td, *J* = 12.9, 8.7 Hz, H-5b), 8.73 (1H, s, H-11), 8.15 (1H, s, H-18), 1.47 (3H, s, H-20), 2.99 (1H, bs, H-21), 3.94 (2H, m, H-22), , 6.83 (1H, bs, NH-14); ^{13}C NMR (DMSO-*d*$_6$, 125 MHz) δ_C 146.3 (CH, C-1), 16.7 (CH_2, C-3), 18.2 (CH_2, C-4), 31.1 (CH_2, C-5), 125.4 (CH, C-11), 123.5 (CH, C-18), 32.6 (CH_3, C-20), 49.3 (CH_2, C-21), 45.0 (CH_2, C-22); MS, ^1H NMR and ^{13}C NMR data are identical with literature values [20,21].

Adociaquinone A (**3**): yellow solid; UV (CH_3CN) λ_{max} 243, 305 nm; LRESIMS *m/z* 424 $[M+H]^+$; HRESIMS *m/z* 424.1284 $[M+H]^+$ (calcd for $C_{22}H_{18}NO_6S$, 424.0855); ^1H NMR (DMSO-*d*$_6$, 500 MHz) δ_H 7.99 (1H, s, H-1), 2.85 (1H, dd, *J* = 16.9, 7.8 Hz, H-3a), 2.59 (1H, m, H-3b), 2.22 (1H, m, H-4a), 2.07 (1H, m, H-4b), 2.59 (1H, m, H-5a), 1.64 (1H, td, *J* = 12.9, 4.2 Hz, H-5b), 8.67 (1H, s, H-11), 8.25 (1H, s, H-18), 1.43 (3H, s, H-20), 3.91 (2H, m, H-21), 3.30 (1H, H-22, overlap with residual H_2O); MS and ^1H NMR data are identical with literature values [20,21].

14-hydroxymethylxestoquinone (**4**): yellow-orange solid; UV (CH_3CN) λ_{max} 218, 261, 299 nm; LRESIMS *m/z* 349 $[M+H]^+$; HRESIMS *m/z* 349.1058 $[M+H]^+$ (calcd for $C_{21}H_{17}O_5$, 349.1076); ^1H NMR ($CDCl_3$, 500 MHz) δ_H 7.53 (1H, s, H-1), 2.88 (1H, m, H-3a), 2.64 (1H, m, H-3b), 2.27 (1H, m, H-4a), 2.18 (1H, m, H-4b), 2.57 (1H, m, H-5a), 1.73 (1H, m, H-5b), 9.05 (1H, s, H-11), 7.11 (1H, s, H-15), 8.24 (1H, s, H-18), 1.53 (3H, s, H-20), 4.72 (2H, s, H-21); ^{13}C NMR ($CDCl_3$, 125 MHz) δ_C 144.6 (CH, C-1), 121.4 (C, C-2), 16.8 (CH_2, C-3), 18.3 (CH_2, C-4), 31.1 (CH_2, C-5), 37.3 (C, C-6), 147.1 (C, C-7), 144.1 (C, C-8), 138.1 (C, C-10), 126.9 (CH, C-11), 133.2 (C, C-12), 183.0 (C, C-13), 149.1 (C, C-14), 134.2 (CH, C-15), 185.2 (C, C-16), 128.9 (C, C-17), 123.0 (CH,

C-18), 156.0 (C, C-19), 32.3 (CH_3, C-20). 59.9 (CH_2, C-21); MS, ^{1}H NMR and ^{13}C NMR data are identical with literature values [22].
15-hydroxymetyhlxestoquinone (**5**): yellow solid; UV (CH_3CN) λ_{max} 220, 263, 298 nm; LRESIMS *m/z* 349 $[M+H]^+$; HRESIMS *m/z* 349.1058 $[M+H]^+$ (calcd for $C_{21}H_{17}O_5$, 349.1076); ^{1}H NMR ($CDCl_3$, 500 MHz) δ_H 7.55 (1H, s, H-1), 2.90 (1H, dd, *J* = 16.9, 7.8 Hz, H-3a), 2.68 (1H, m, H-3b), 2.28 (1H, m, H-4a), 2.15 (1H, m, H-4b), 2.57 (1H, m, H-5a), 1.76 (1H, m, H-5b), 9.02 (1H, s, H-11), 7.10 (1H, s, H-14), 8.25 (1H, s, H-18), 1.54 (3H, s, H-20), 4.74 (2H, s, H-21); MS and ^{1}H NMR data are identical with literature values [22].

2:1 mixture of 14- and 15-methoxyxestoquinone (**6**): light yellow solid; UV (CH_3CN) λ_{max} 223, 273, 285 nm; LRESIMS *m/z* 349 $[M+H]^+$; HRESIMS *m/z* 349.1353 $[M+H]^+$ (calcd for $C_{21}H_{17}O_5$, 349.1076); ^{1}H NMR ($CDCl_3$, 500 MHz) δ_H 7.53 (1H, s, H-1), 2.89 (1H, dd, *J* = 16.9, 7.8 Hz, H-3a), 2.56 (1H, m, H-3b), 2.28 (1H, m, H-4a), 2.17 (1H, m, H-4b), 2.65 (1H, m, H-5a), 1.75 (1H, m, H-5b), 9.08 (1H, s, H-11, major), 9.04 (1H, s, H-11, minor), 6.24 (1H, s, H-15, major), 6.25 (1H, s, H-14, minor), 8.25 (1H, s, H-18, major), 8.28 (1H, s, H-18, minor), 1.5.3 (3H, s, H-20), 3.39 (3H, s, C-21); ^{13}C NMR ($CDCl_3$, 125 MHz) δ_C 144.8 (CH, C-1), 16.8 (CH_2, C-3), 18.3 (CH_2, C-4), 31.1 (CH_2, C-5), 127.3 (CH, C-11, major), 126.7 (CH, C-11, minor), 110.4 (CH, C-15, major), 110.1 (CH, C-14, minor), 123.1 (CH, C-18, major), 123.8 (CH, C-18, minor), 32.4 (CH_3, C-20), 56.5 (CH_3, C-21); MS, ^{1}H NMR and ^{13}C NMR data are identical with literature values [20,23].

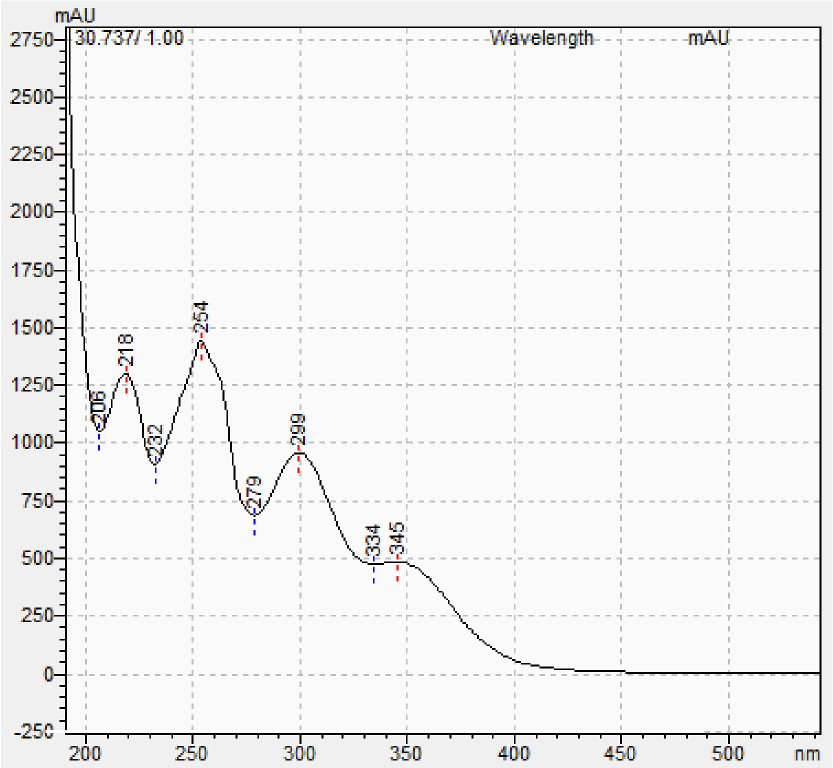

Figure S2. UV profile of xestoquinone (**1**) in CH_3CN

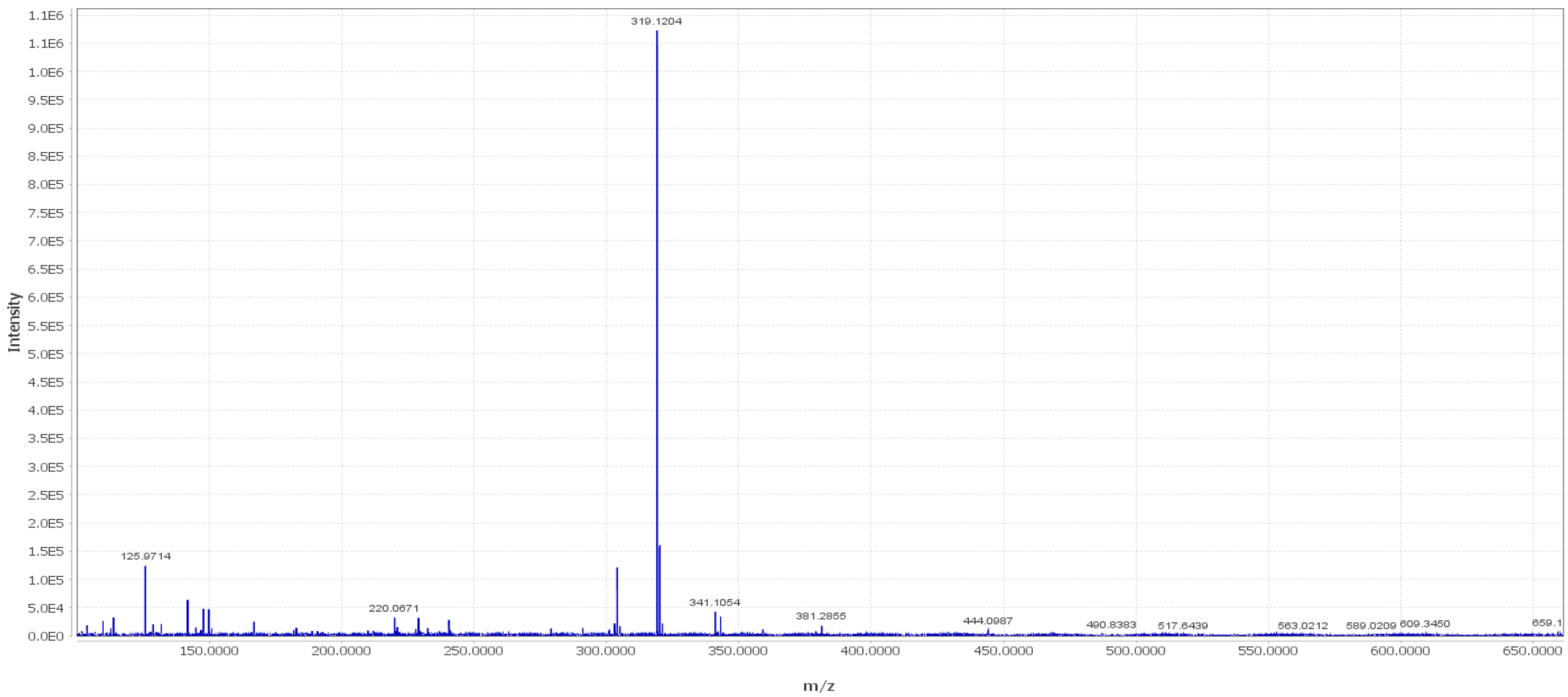

Figure S3. HRESIMS spectrum of xestoquinone (**1**) in positive mode

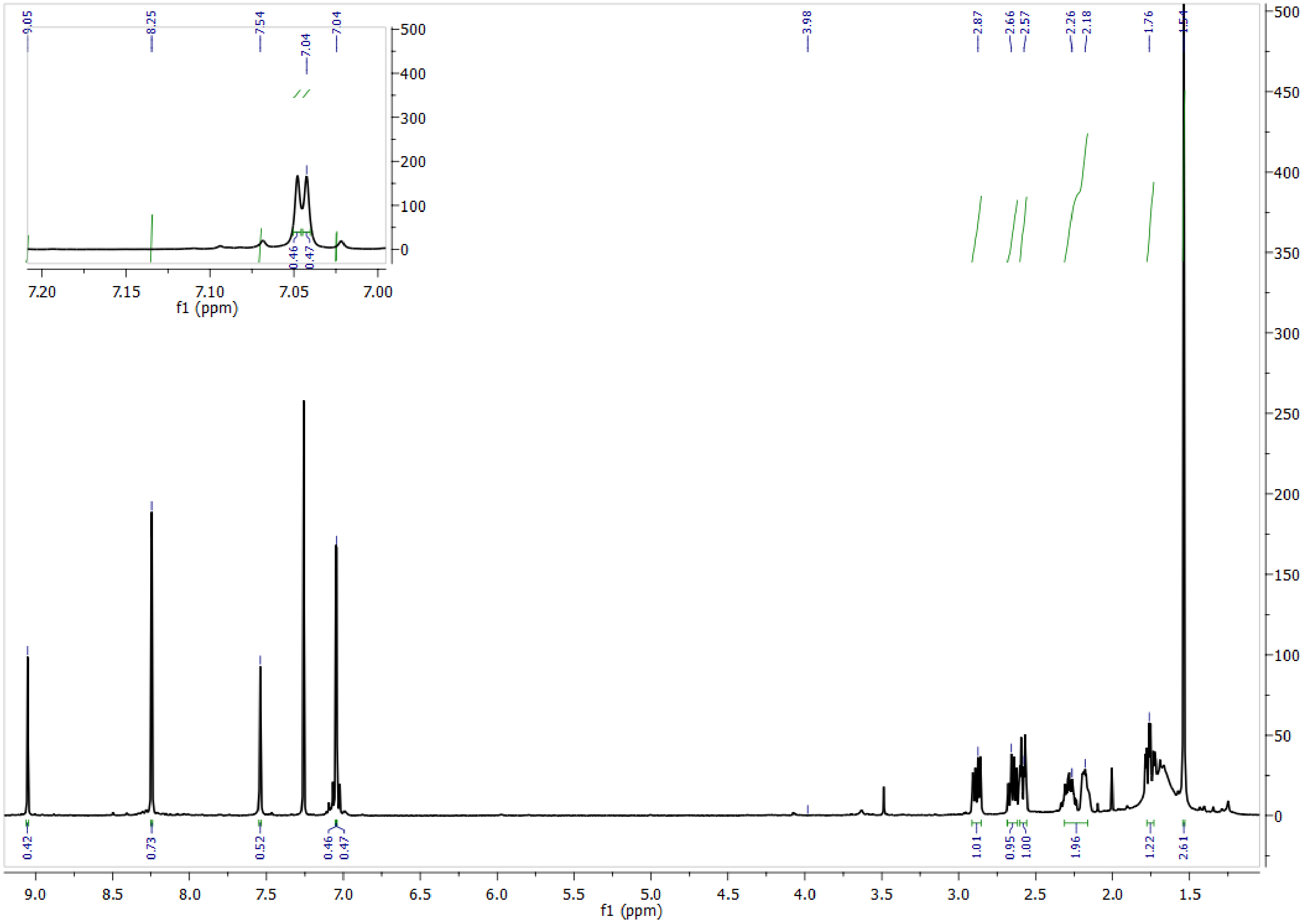

Figure S4. ^{1}H NMR spectrum of xestoquinone (**1**) in $CDCl_3$ (500 MHz)

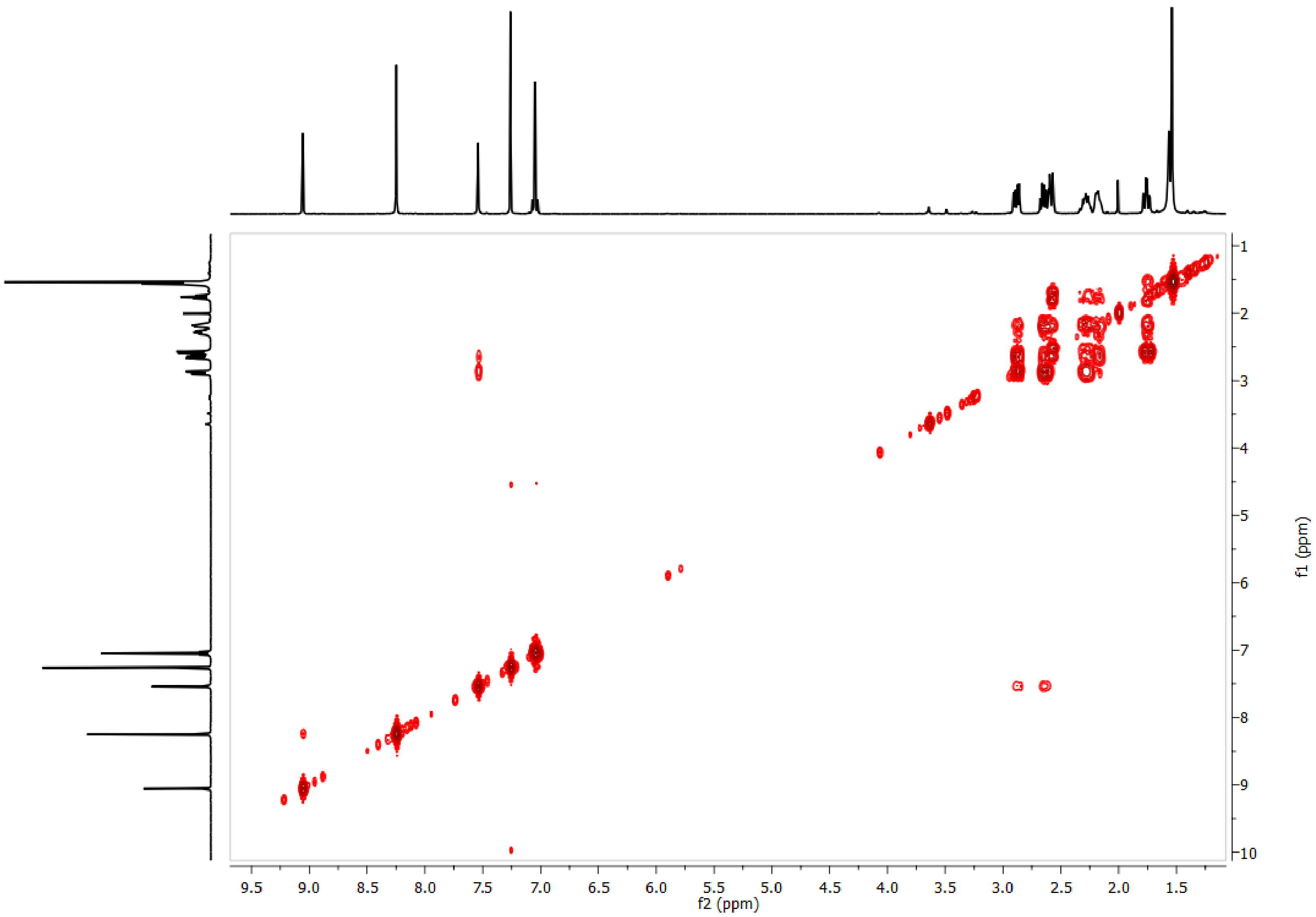

Figure S5. COSY spectrum of xestoquinone (**1**) in $CDCl_3$ (500 MHz)

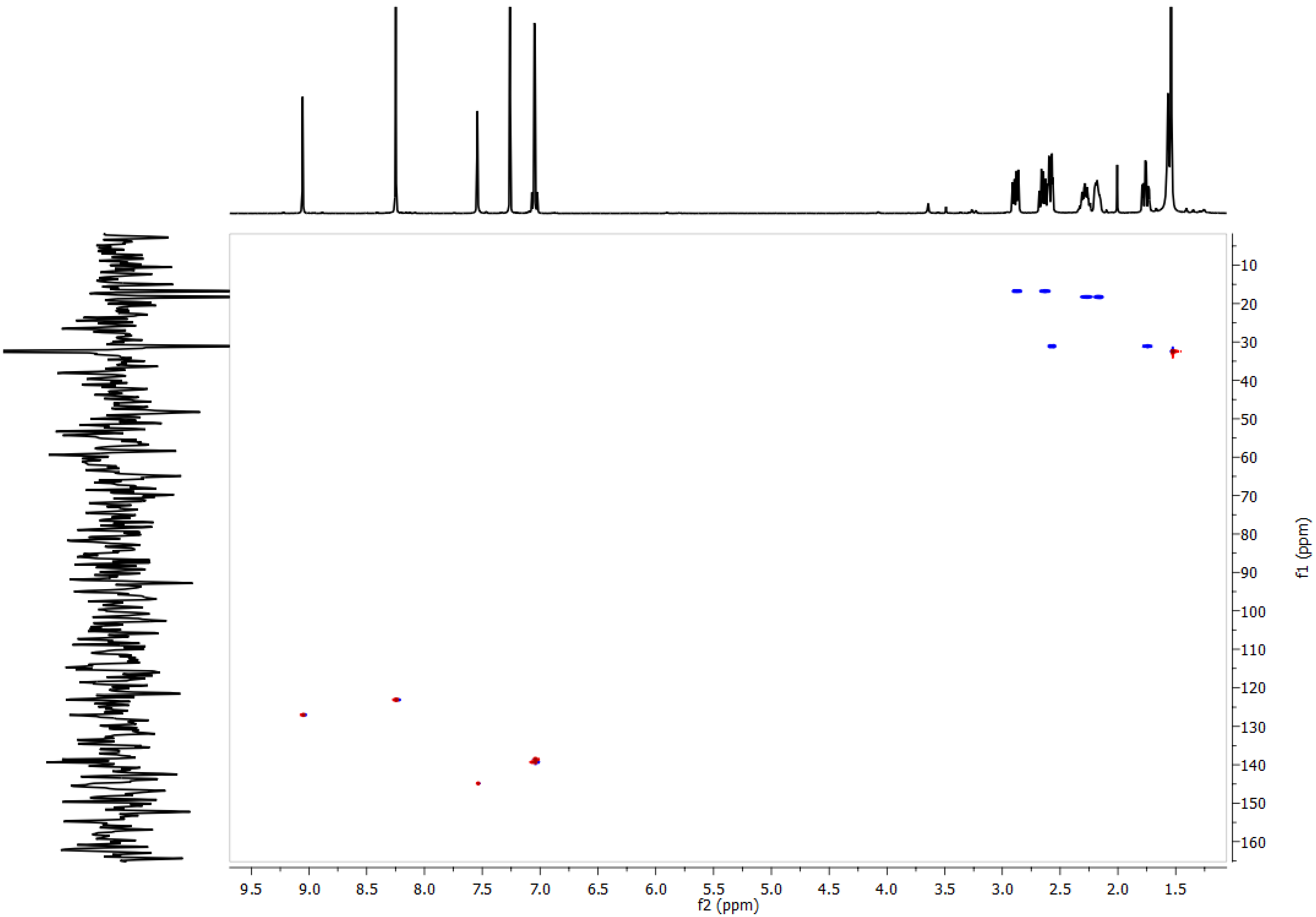

Figure S6. HSQC spectrum of xestoquinone (**1**) in $CDCl_3$ (500 MHz)

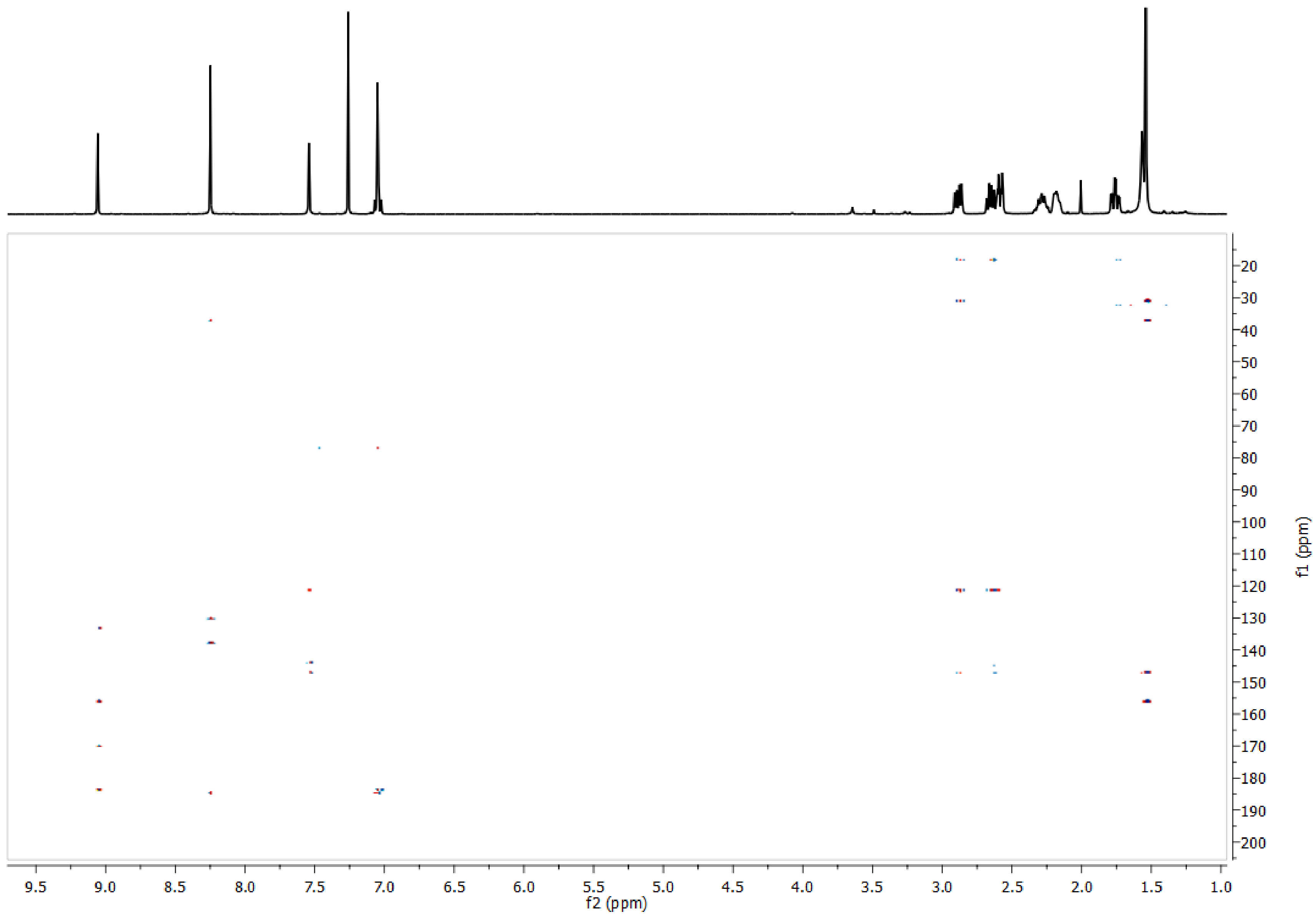

Figure S7. HMBC spectrum of xestoquinone (**1**) in $CDCl_3$ (500 MHz)

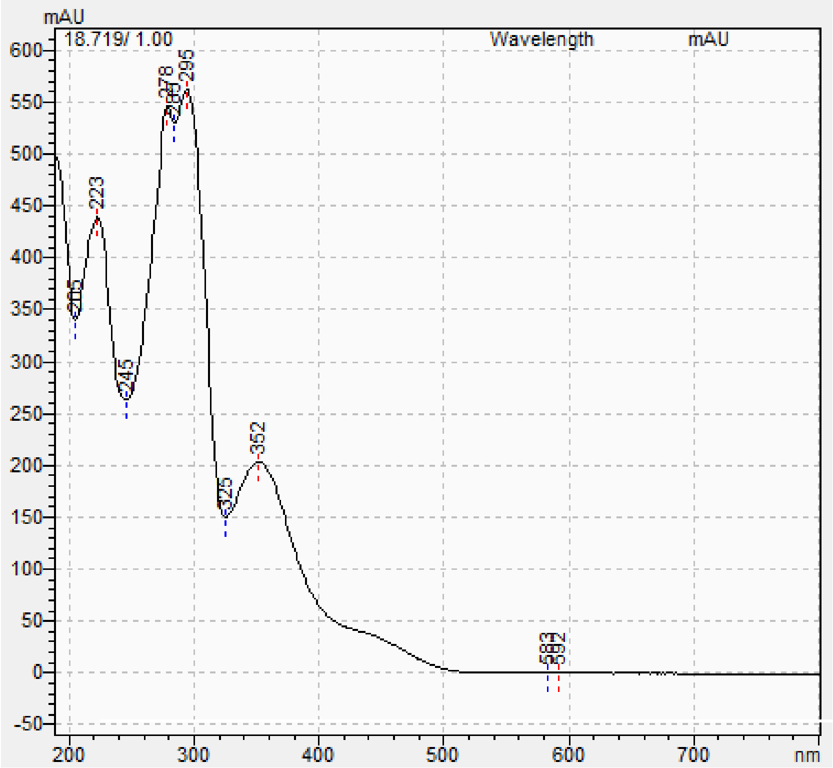

Figure S8. UV profile of adociaquinone B (**2**) in CH_3CN

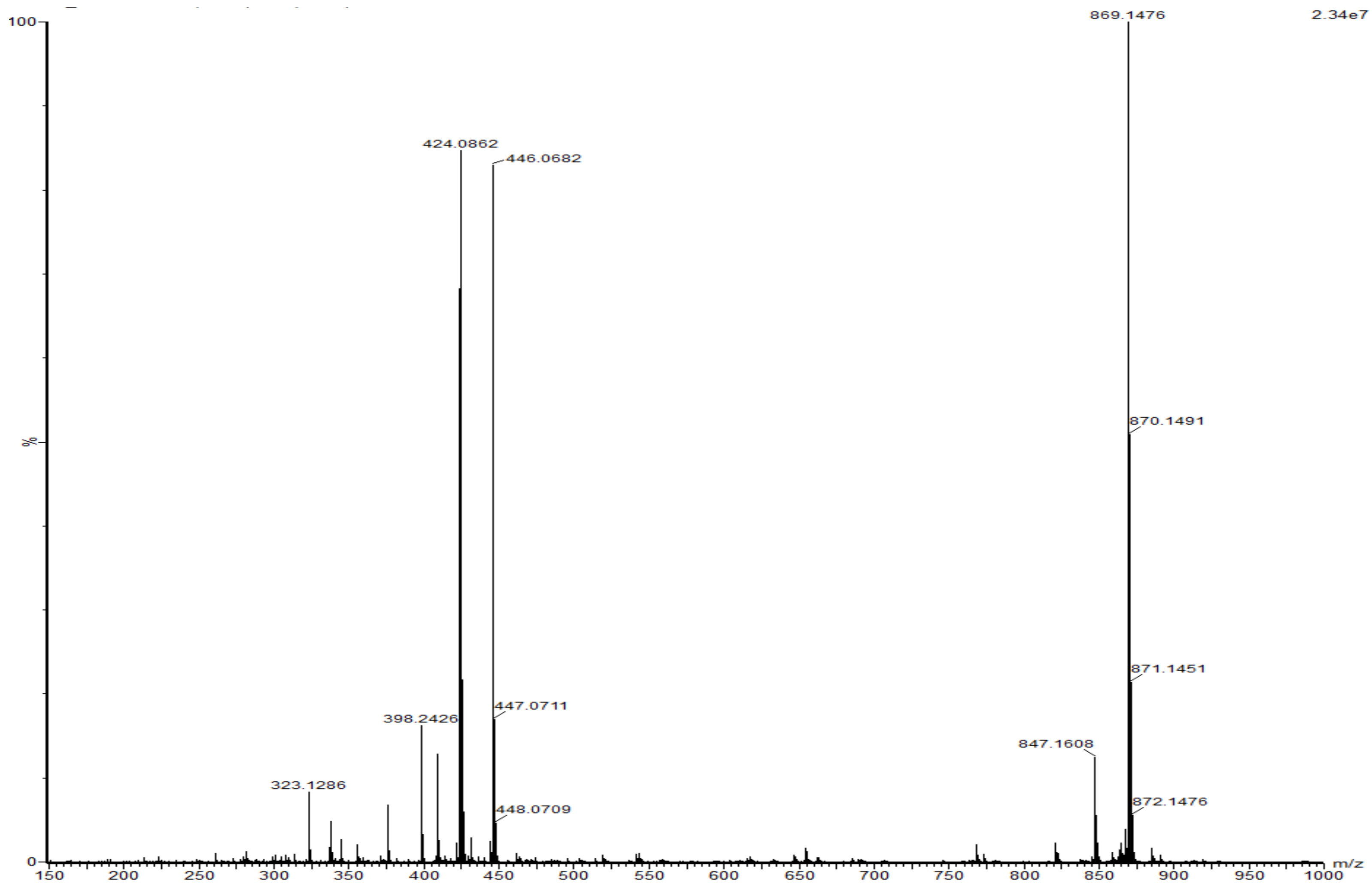

Figure S9. HRESIMS spectrum of adociaquinone B (**2**) in positive mode

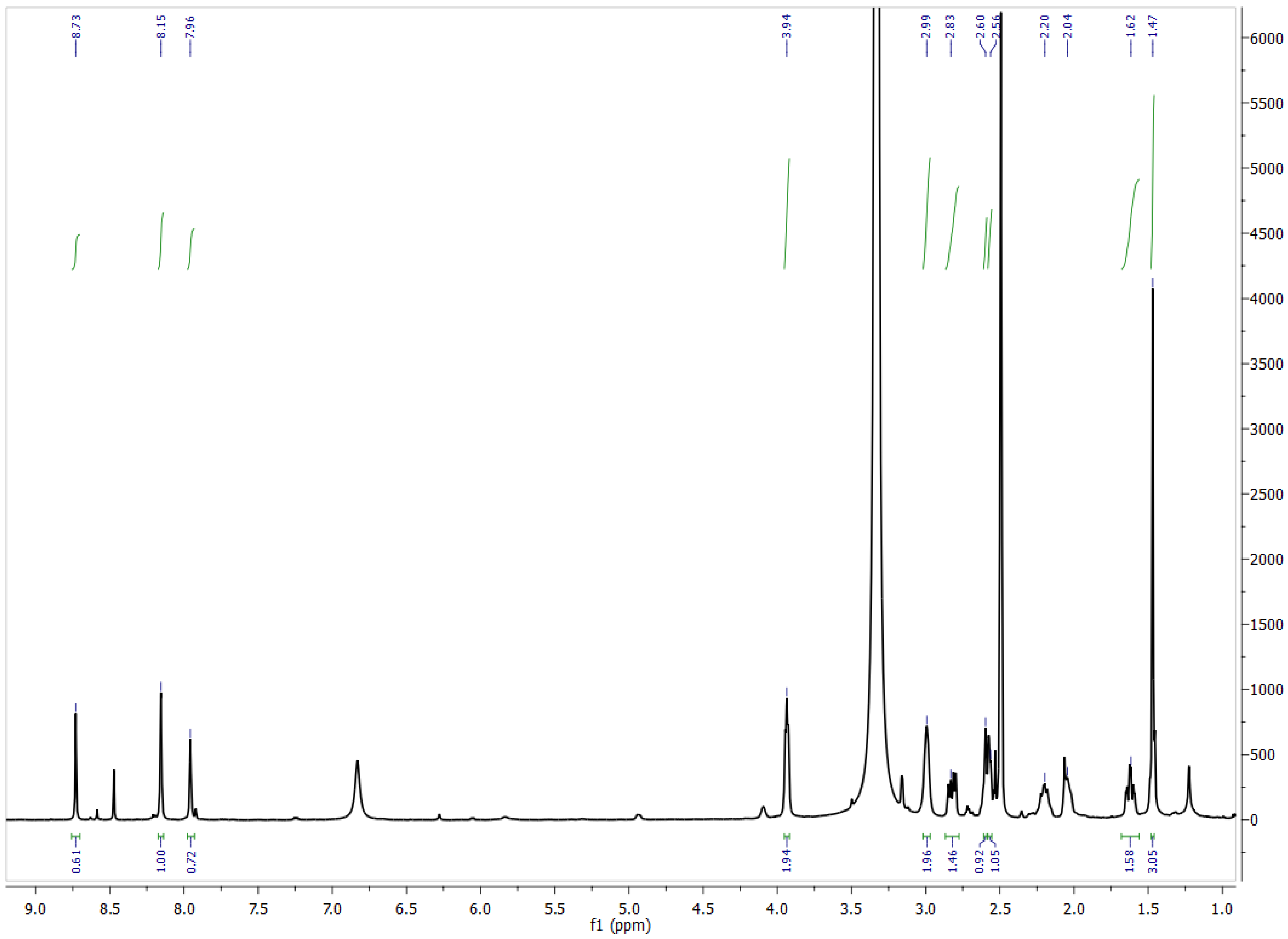

Figure S10. ^{1}H NMR spectrum of adociaquinone B (**2**) in DMSO-d_6 (500 MHz)

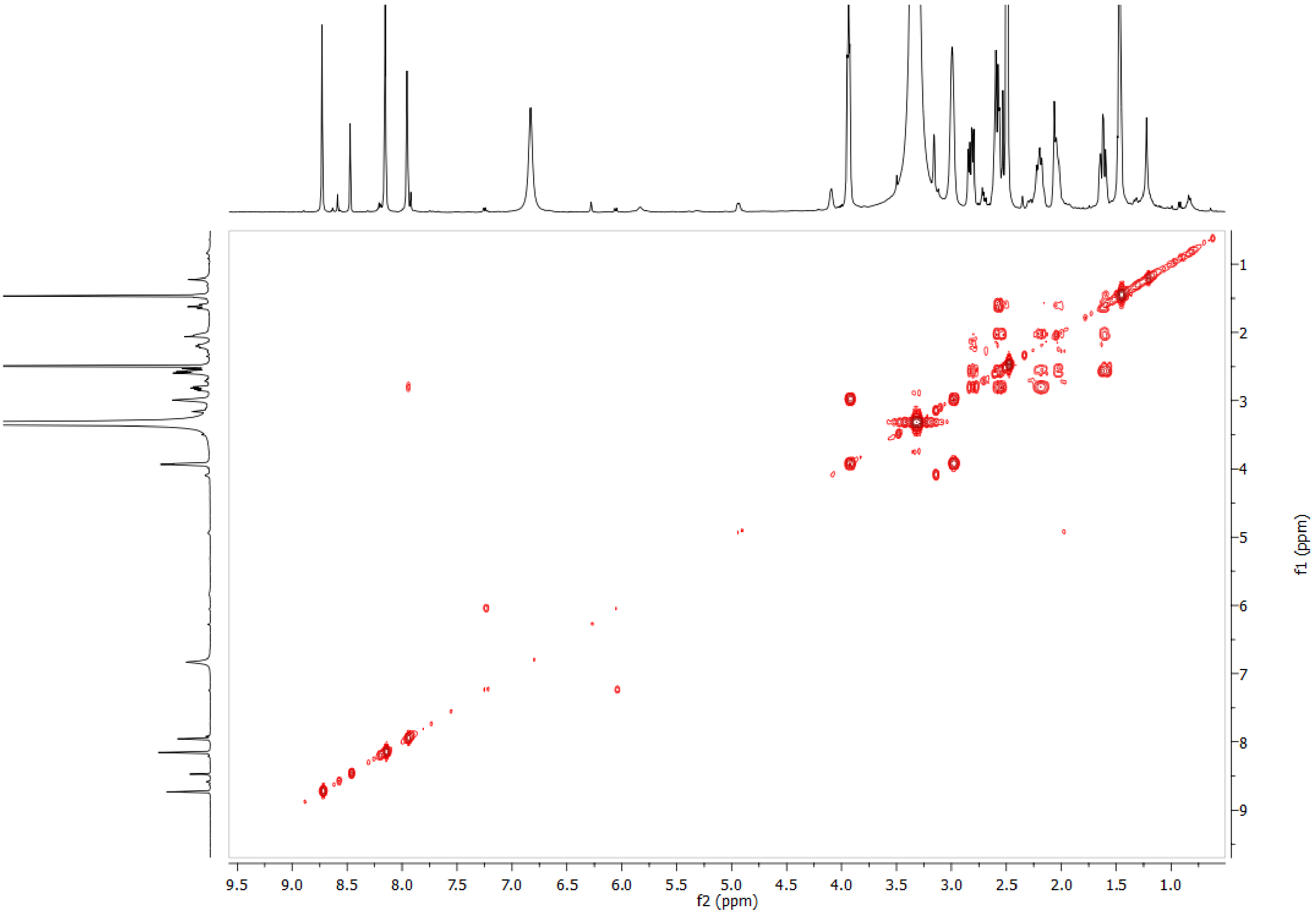

Figure S11. COSY spectrum of adociaquinone B (**2**) in DMSO-d_6 (500 MHz)

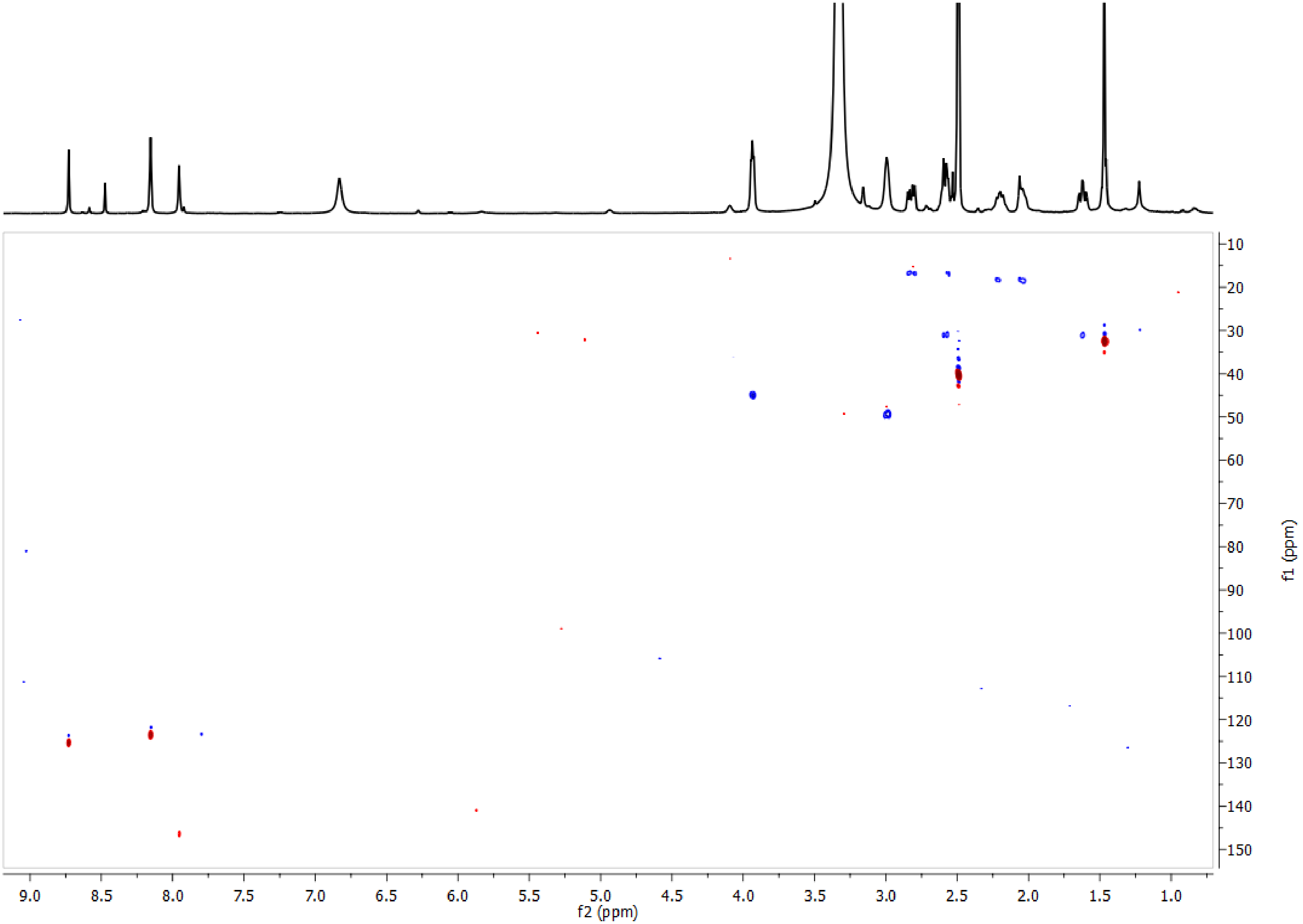

Figure S12. HSQC spectrum of adociaquinone B (**2**) in DMSO-d_6 (500 MHz)

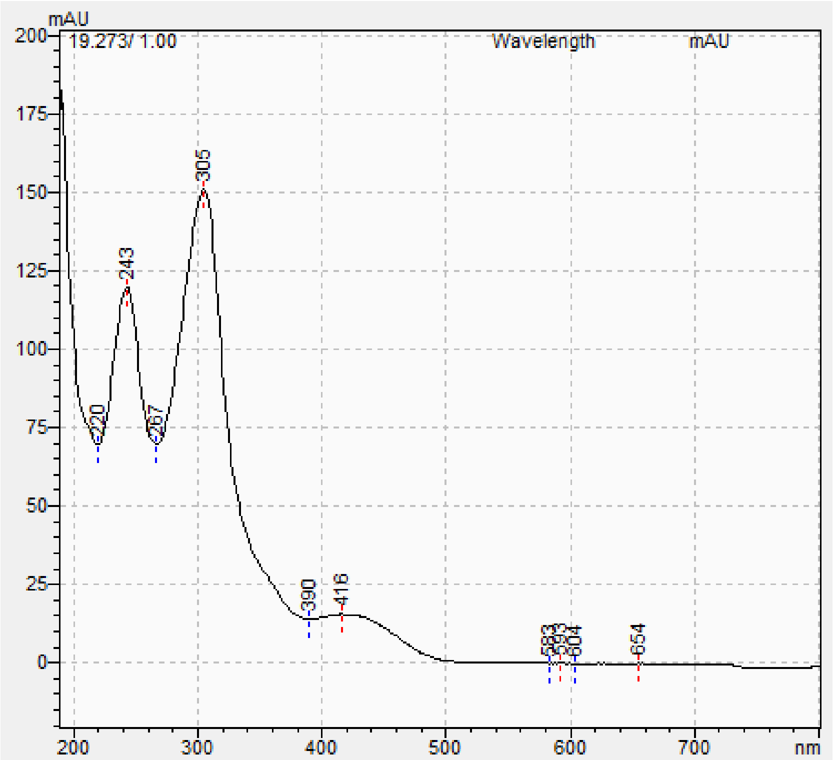

Figure S13. UV profile of adociaquinone A (**3**) in CH_3CN

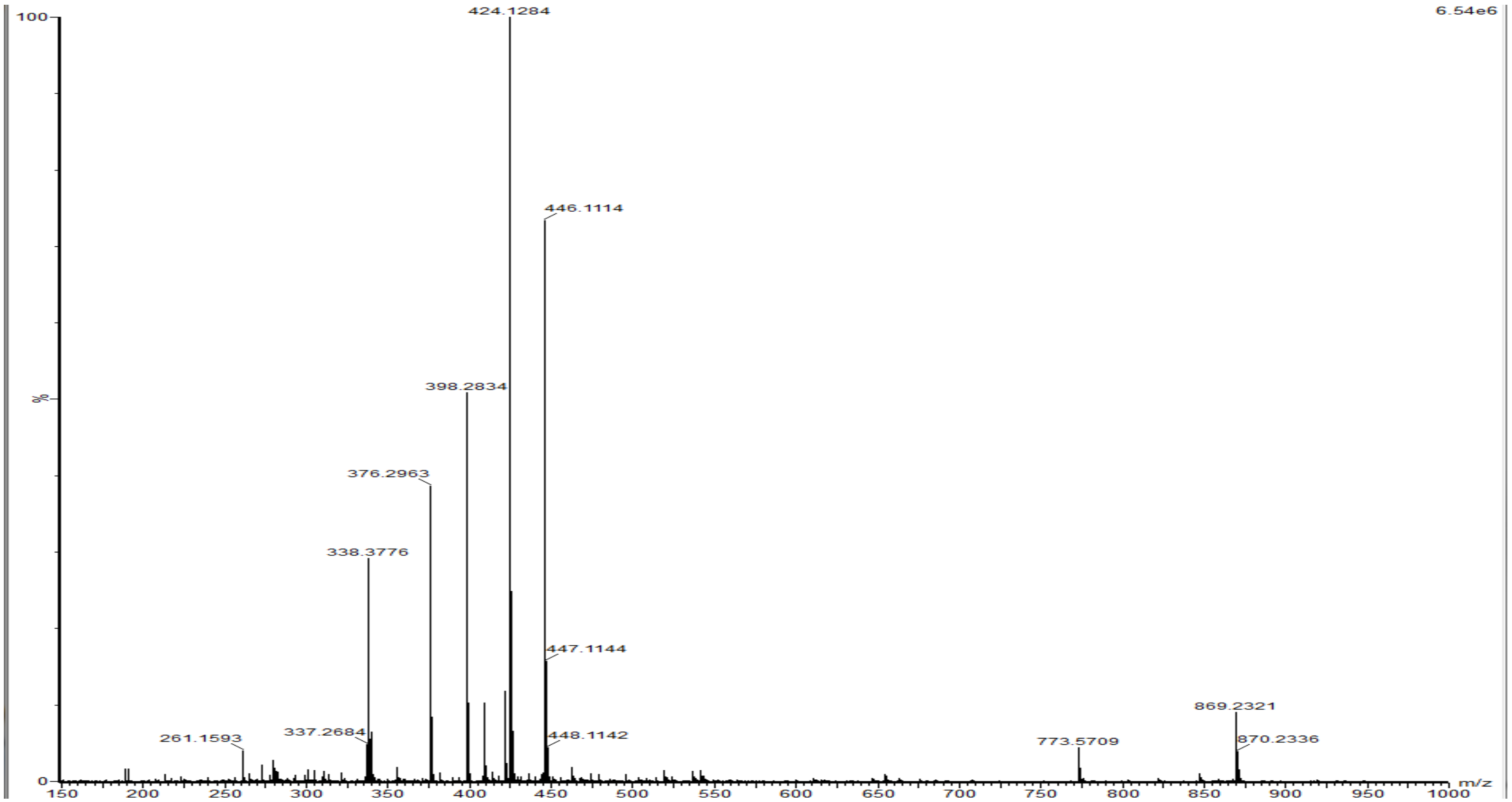

Figure S14. HRESIMS spectrum of adociaquinone A (**3**) in positive mode

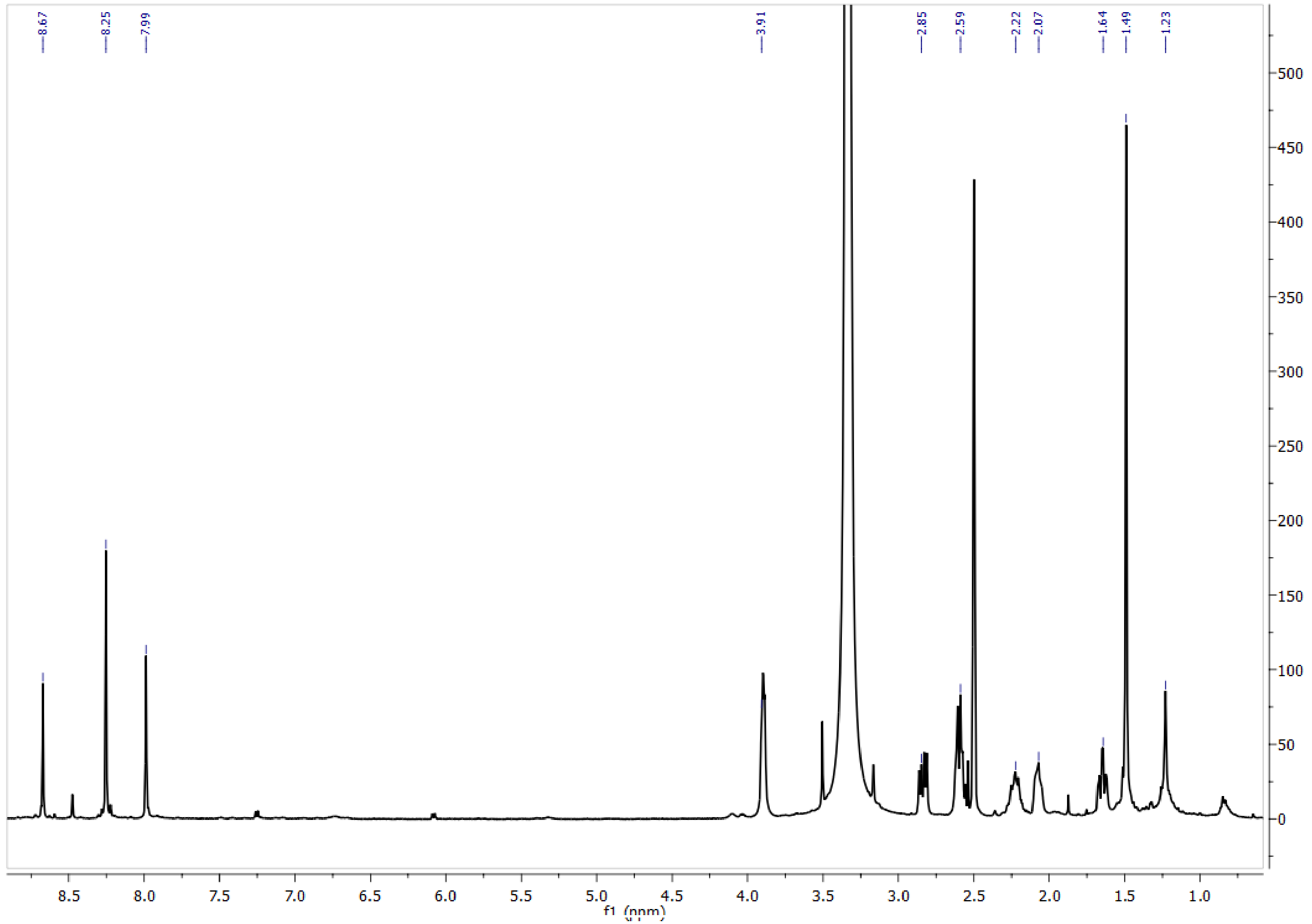

Figure S15. ^{1}H NMR spectrum of adociaquinone A (**3**) in DMSO-d_6 (500 MHz)

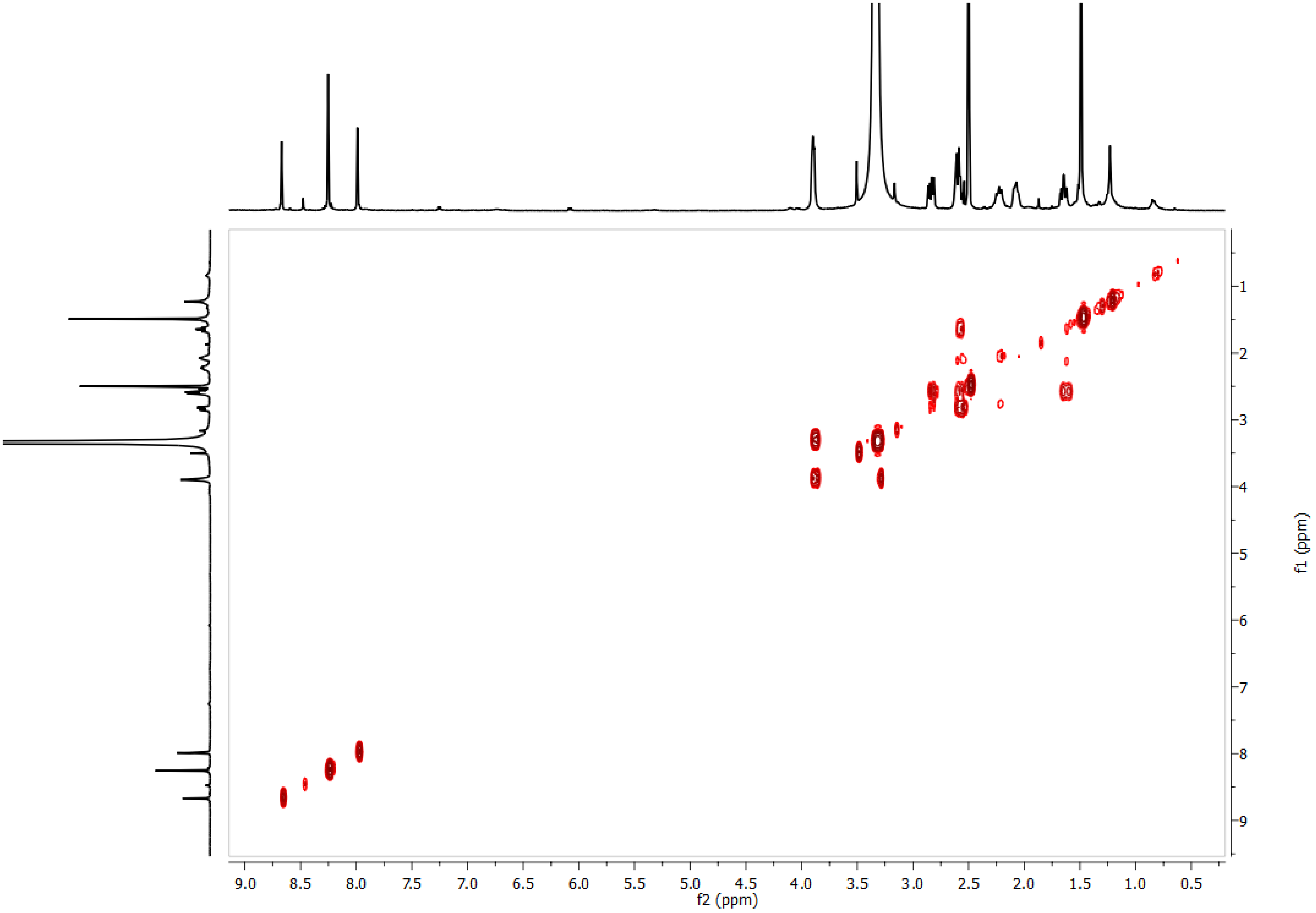

Figure S16. COSY spectrum of adociaquinone A (**3**) in DMSO-d_6 (500 MHz)

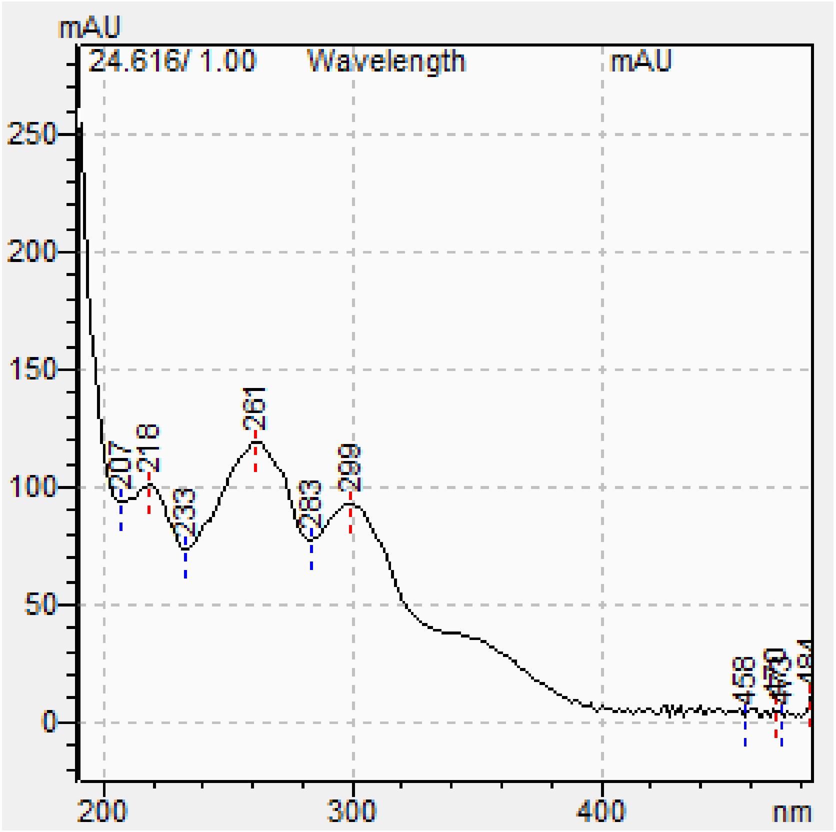

Figure S17. UV profile of 14-hydroxymethylxestoquinone (**4**) in CH_3CN

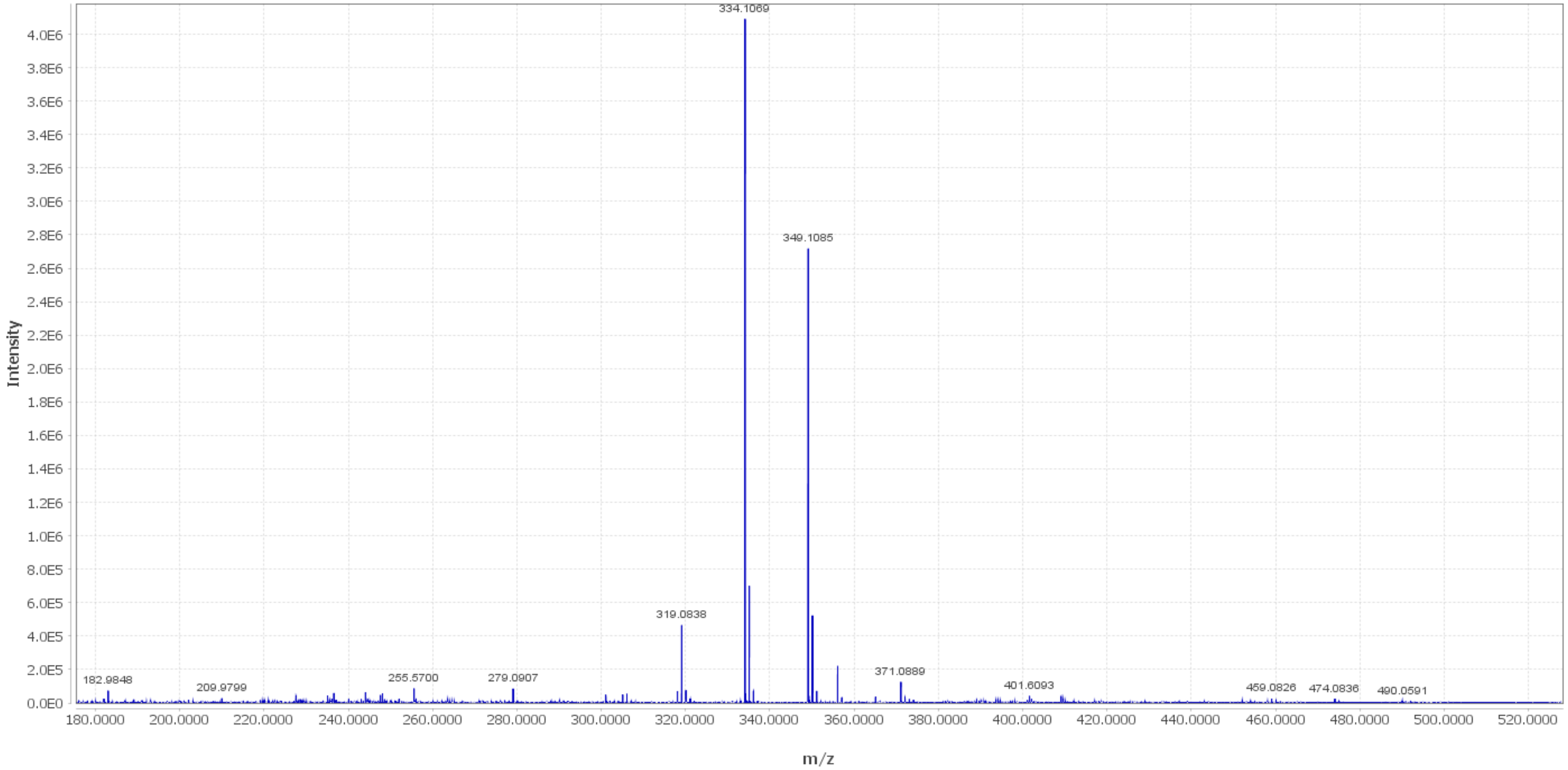

Figure S18. HRESIMS spectrum of 14-hydroxymethylxestoquinone (**4**) in positive mode

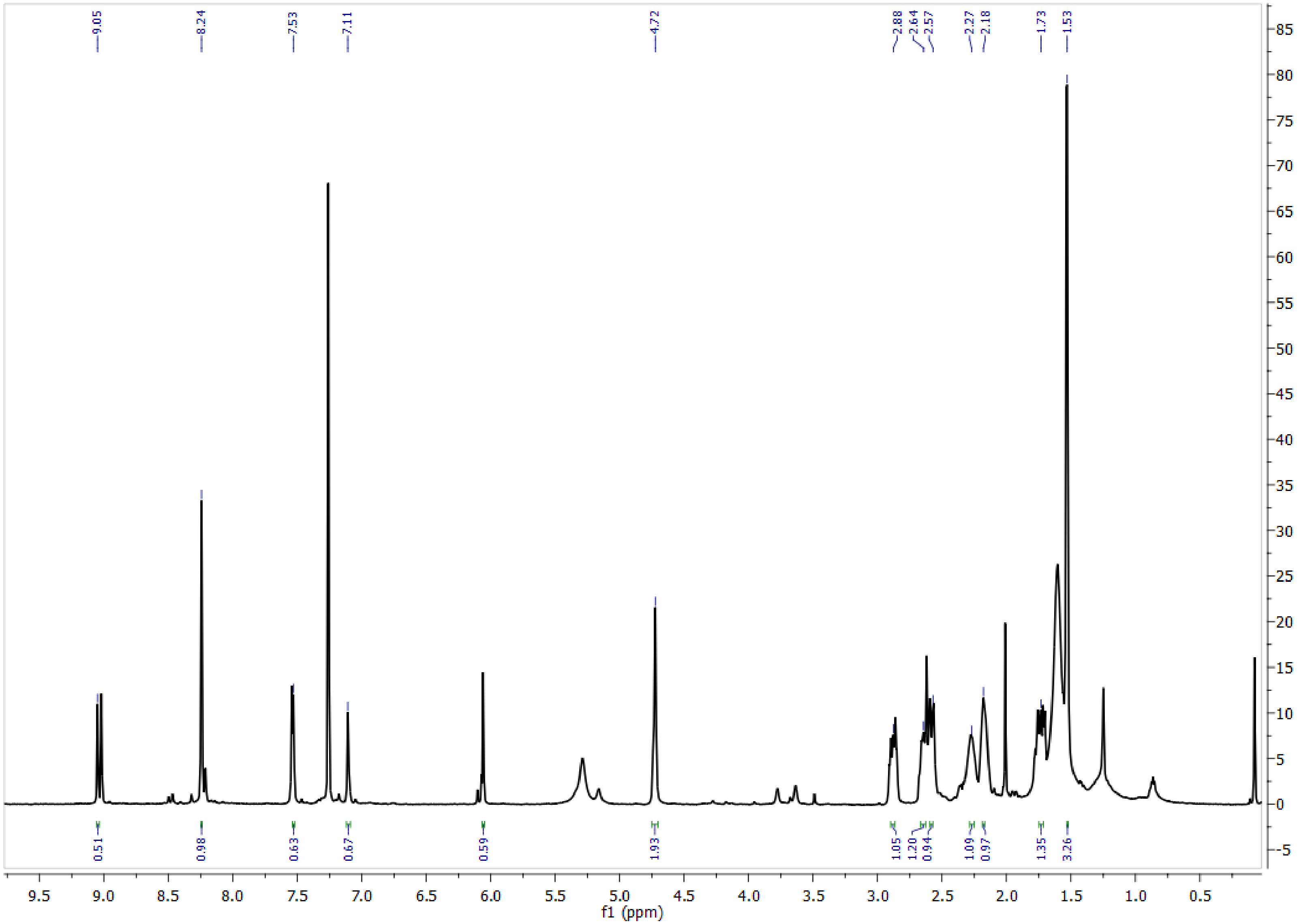

Figure S19. ^{1}H NMR spectrum of 14-hydroxymethylxestoquinone (**4**) in $CDCl_3$ (500 MHz)

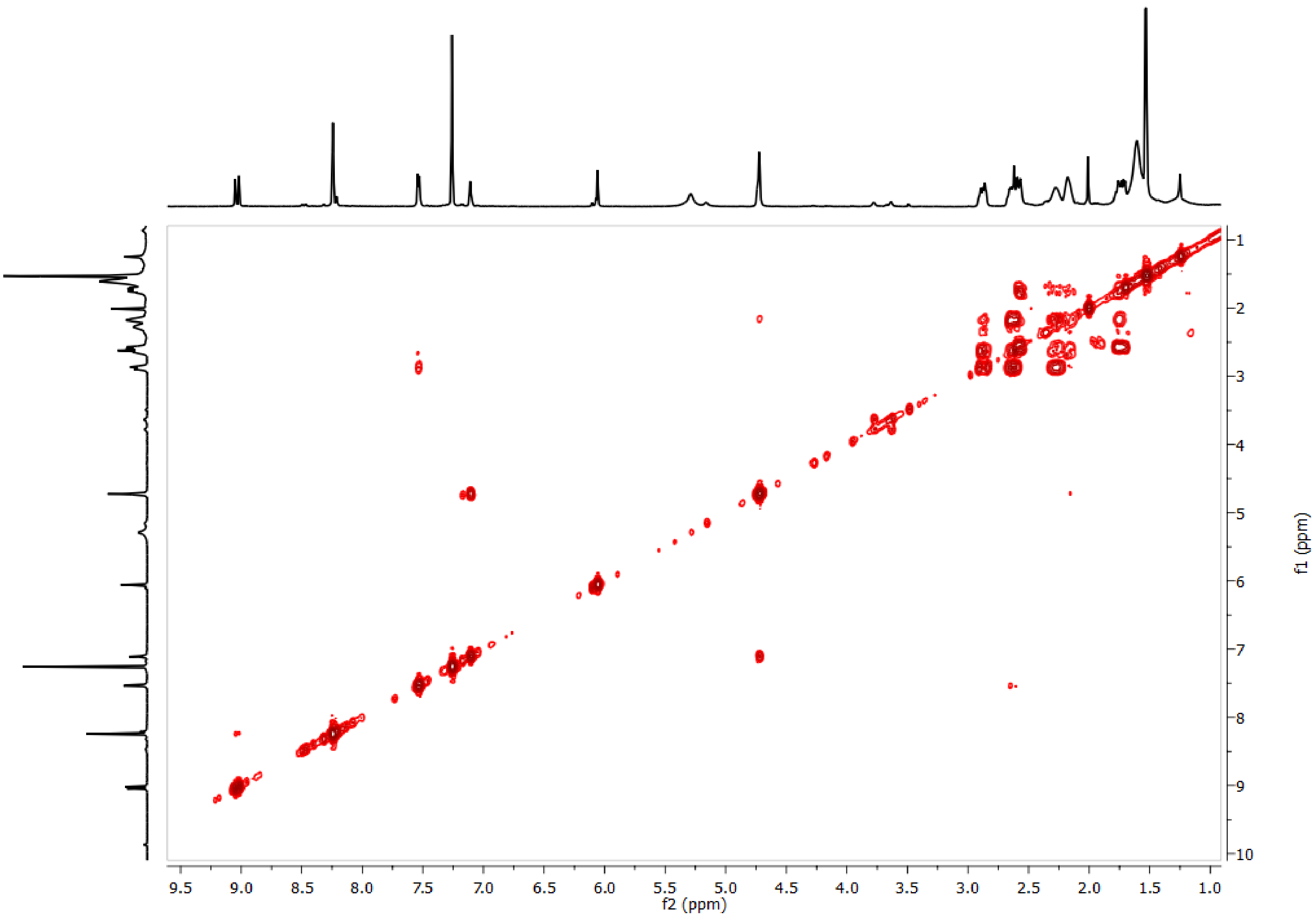

Figure S20. COSY spectrum of 14-hydroxymethylxestoquinone (**4**) in $CDCl_3$ (500 MHz)

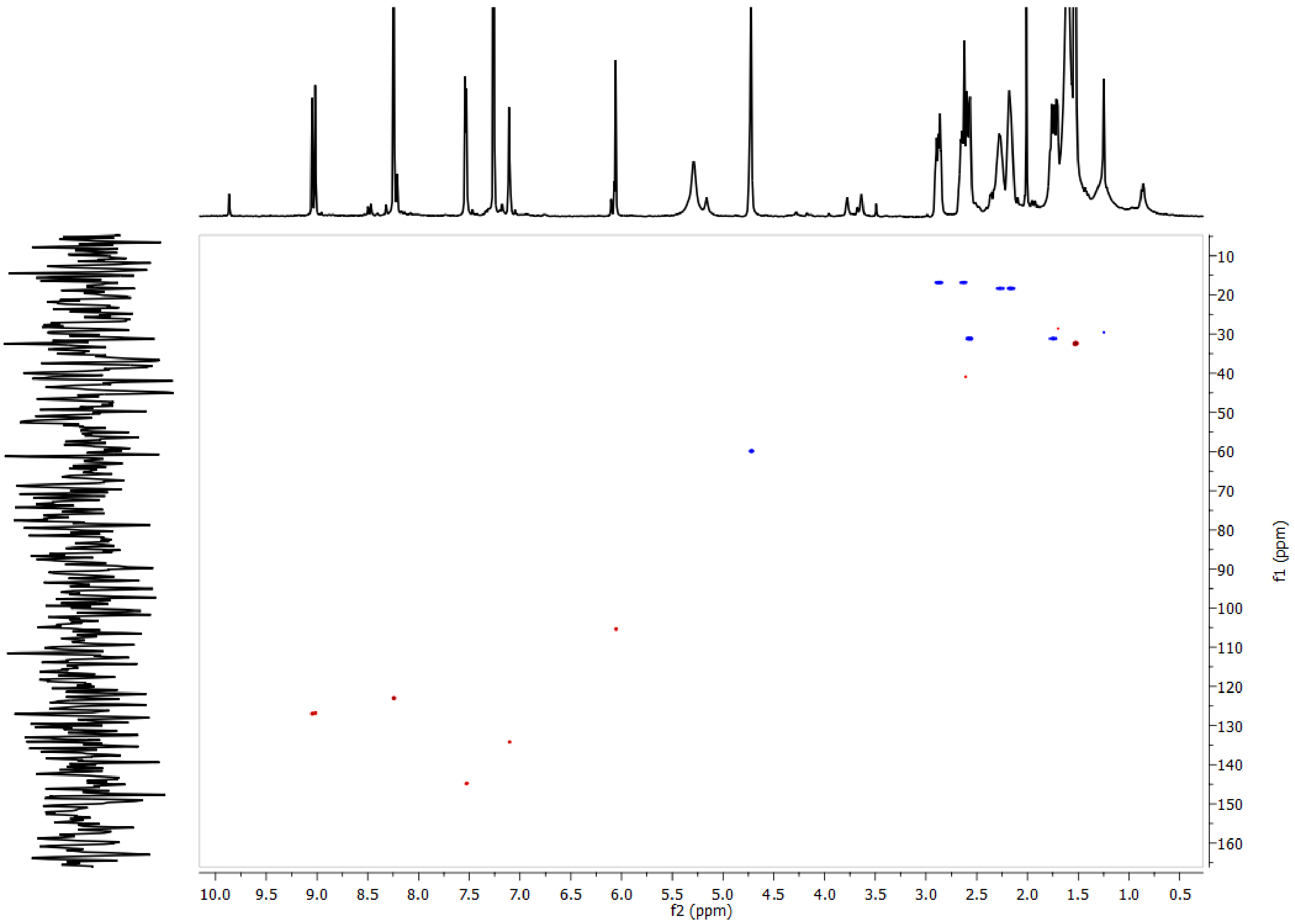

Figure S21. HSQC spectrum of 14-hydroxymethylxestoquinone (**4**) in $CDCl_3$ (500 MHz)

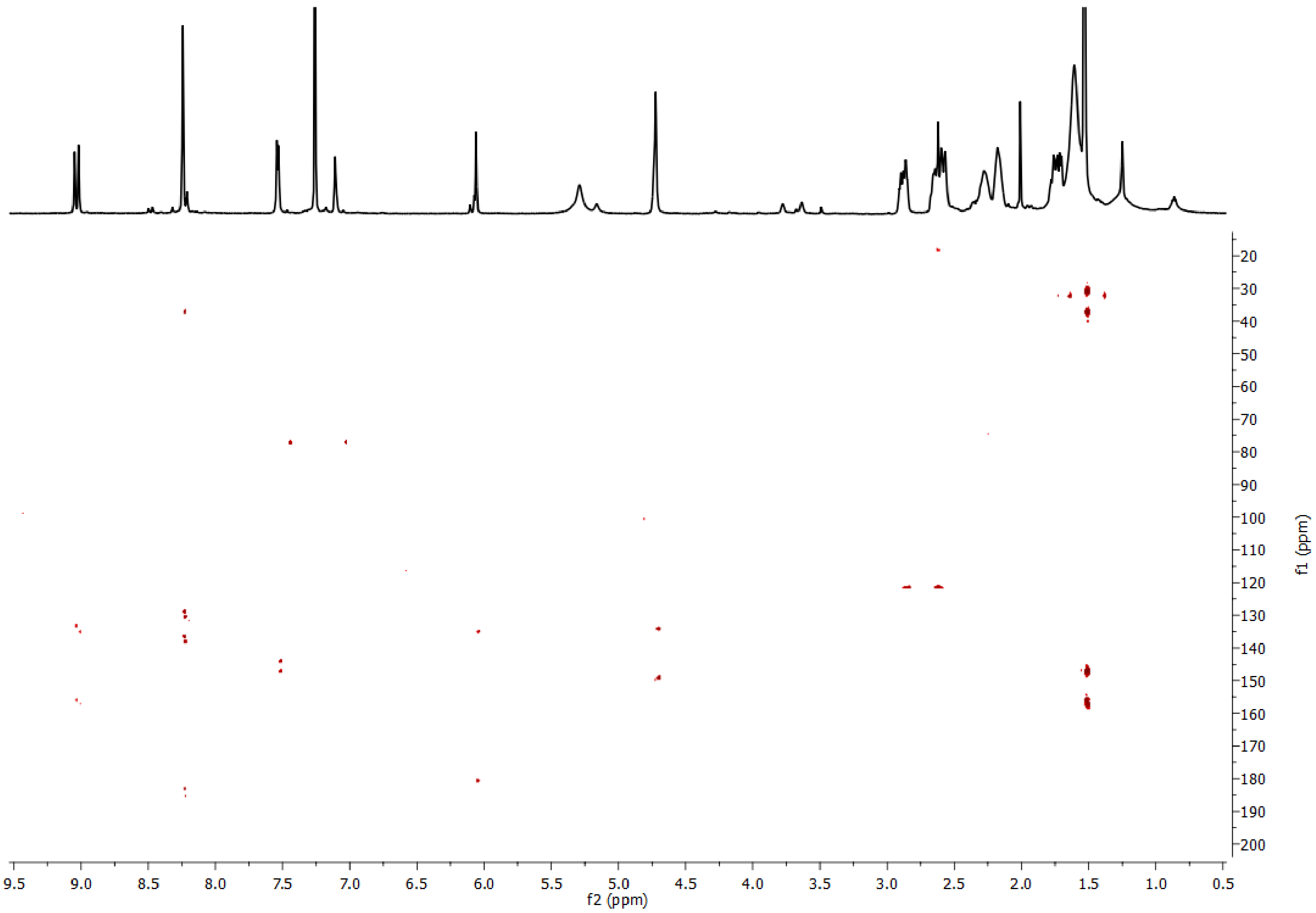

Figure S22. HMBC spectrum of 14-hydroxymethylxestoquinone (**4**) in $CDCl_3$ (500 MHz)

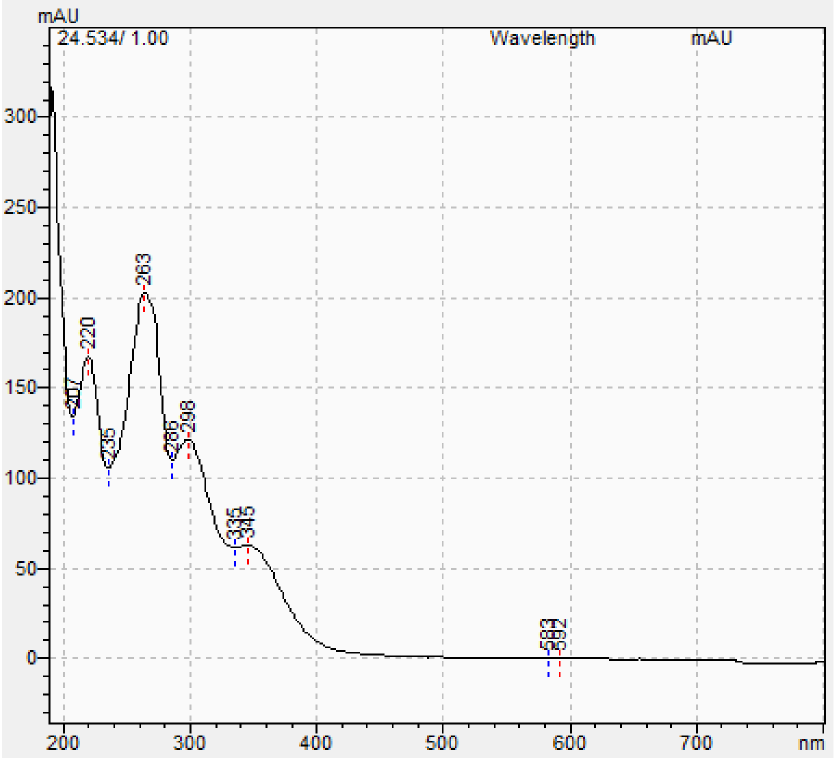

Figure S23. UV profile of 15-hydroxymethylxestoquinone (**5**) in CH_3CN

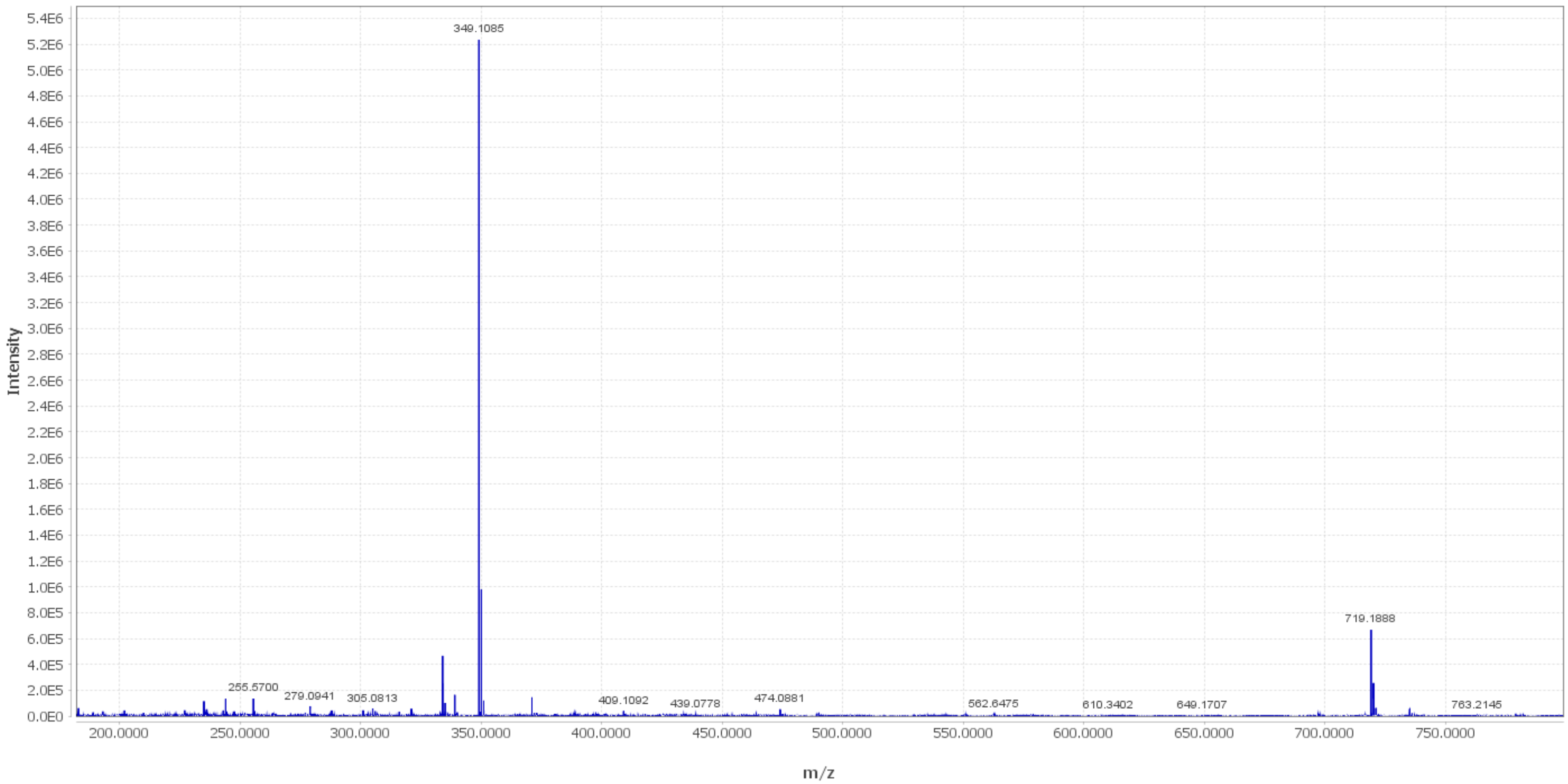

Figure S24. HRESIMS spectrum of 15-hydroxymethylxestoquinone (**5**) in positive mode

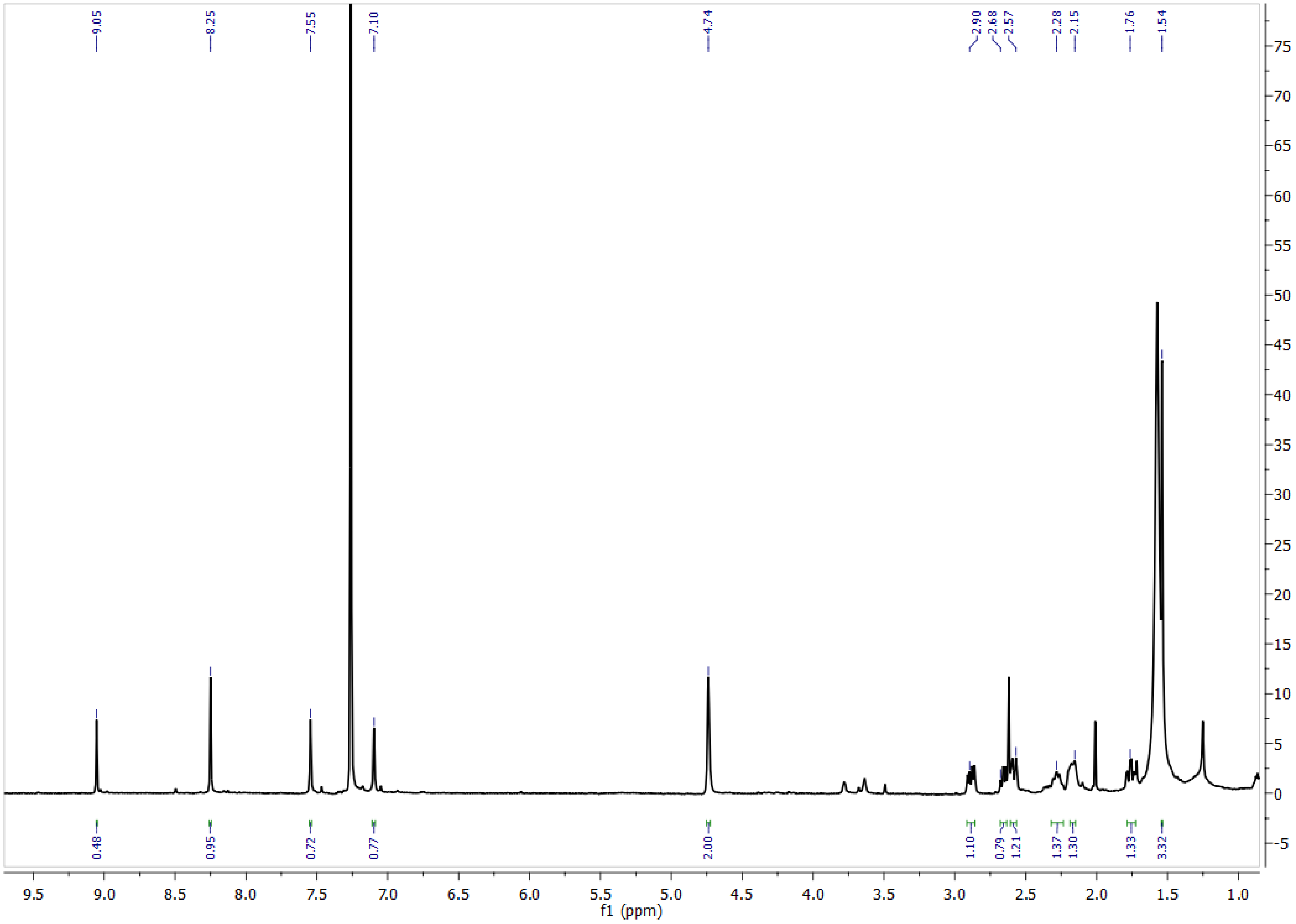

Figure S25. ^{1}H NMR spectrum of 15-hydroxymethylxestoquinone (**5**) in $CDCl_3$ (500 MHz)

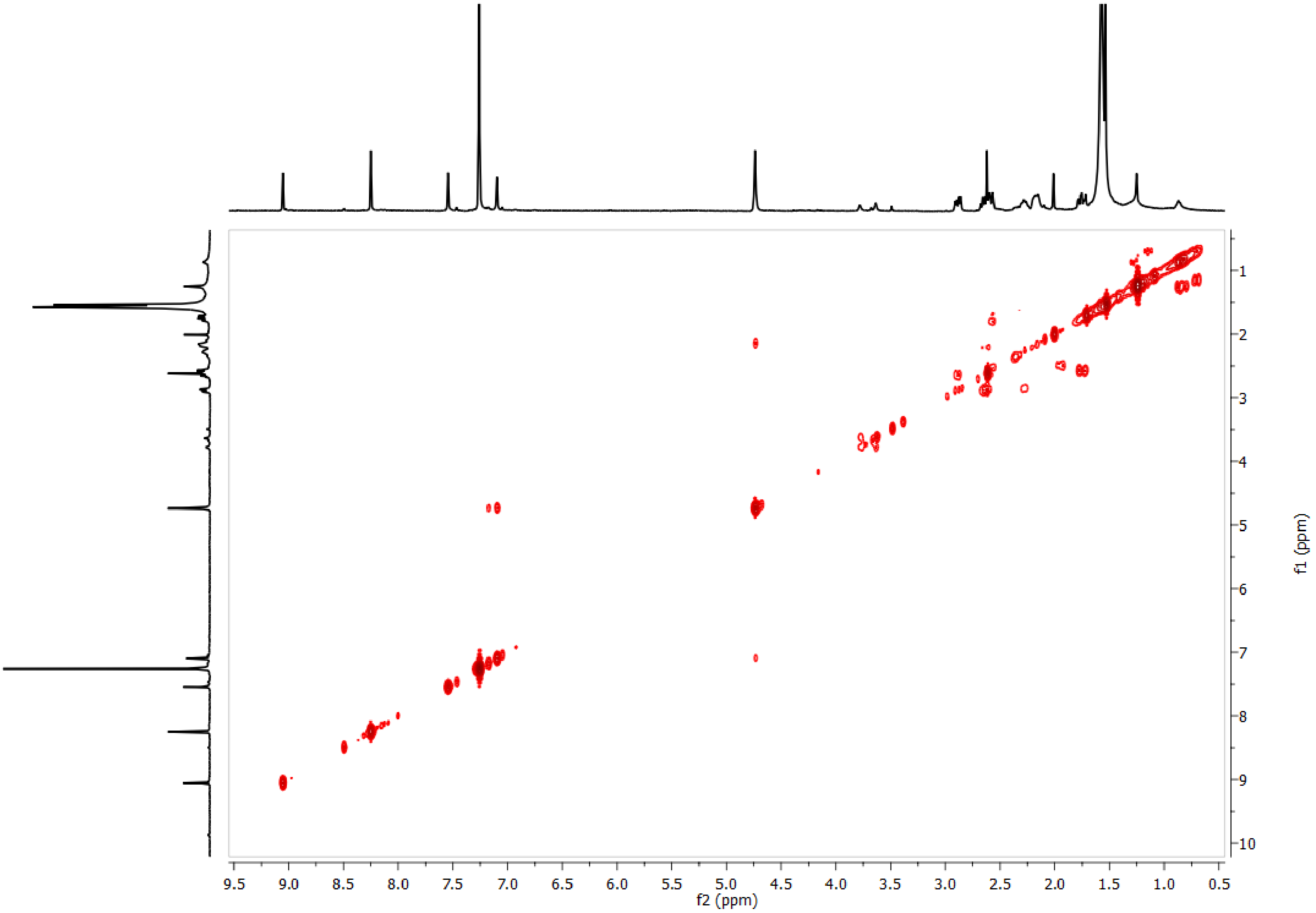

Figure S26. COSY spectrum of 15-hydroxymethylxestoquinone (**5**) in $CDCl_3$ (500 MHz)

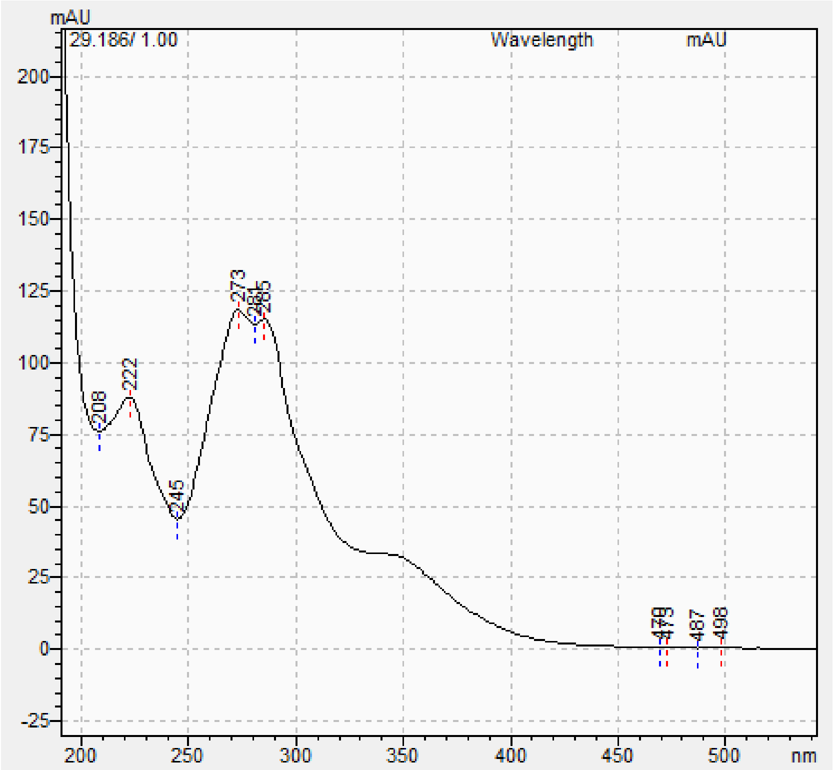

Figure S27. UV profile of 2:1 mixture of 14- and 15-methoxyxestoquinone (**6**) in CH_3CN

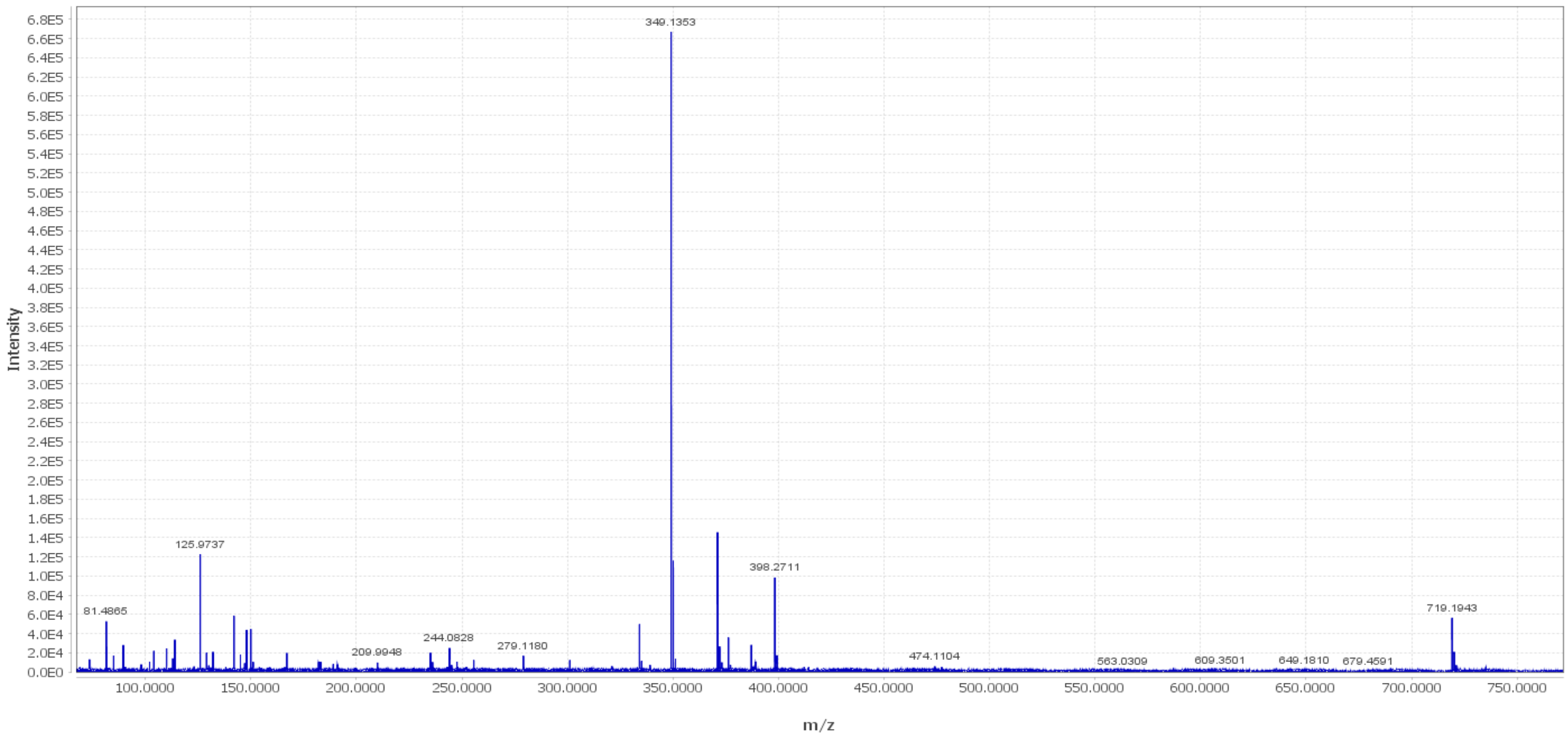

Figure S28. HRESIMS spectrum of 2:1 mixture of 14- and 15-methoxyxestoquinone (**6**) in positive mode

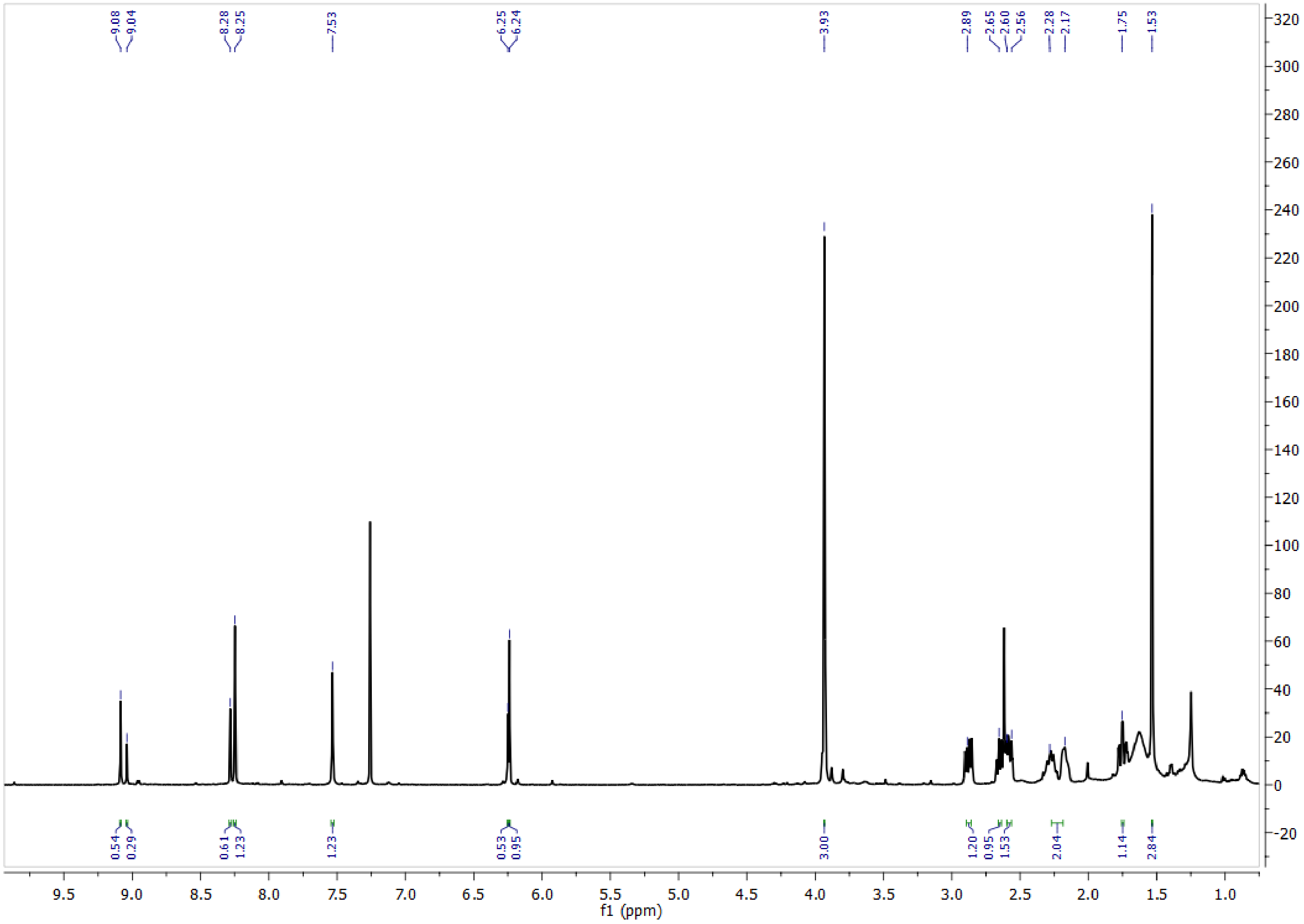

Figure S29. ^{1}H NMR spectrum of 2:1 mixture of 14- and 15-methoxyxestoquinone (**6**) in $CDCl_3$ (500 MHz)

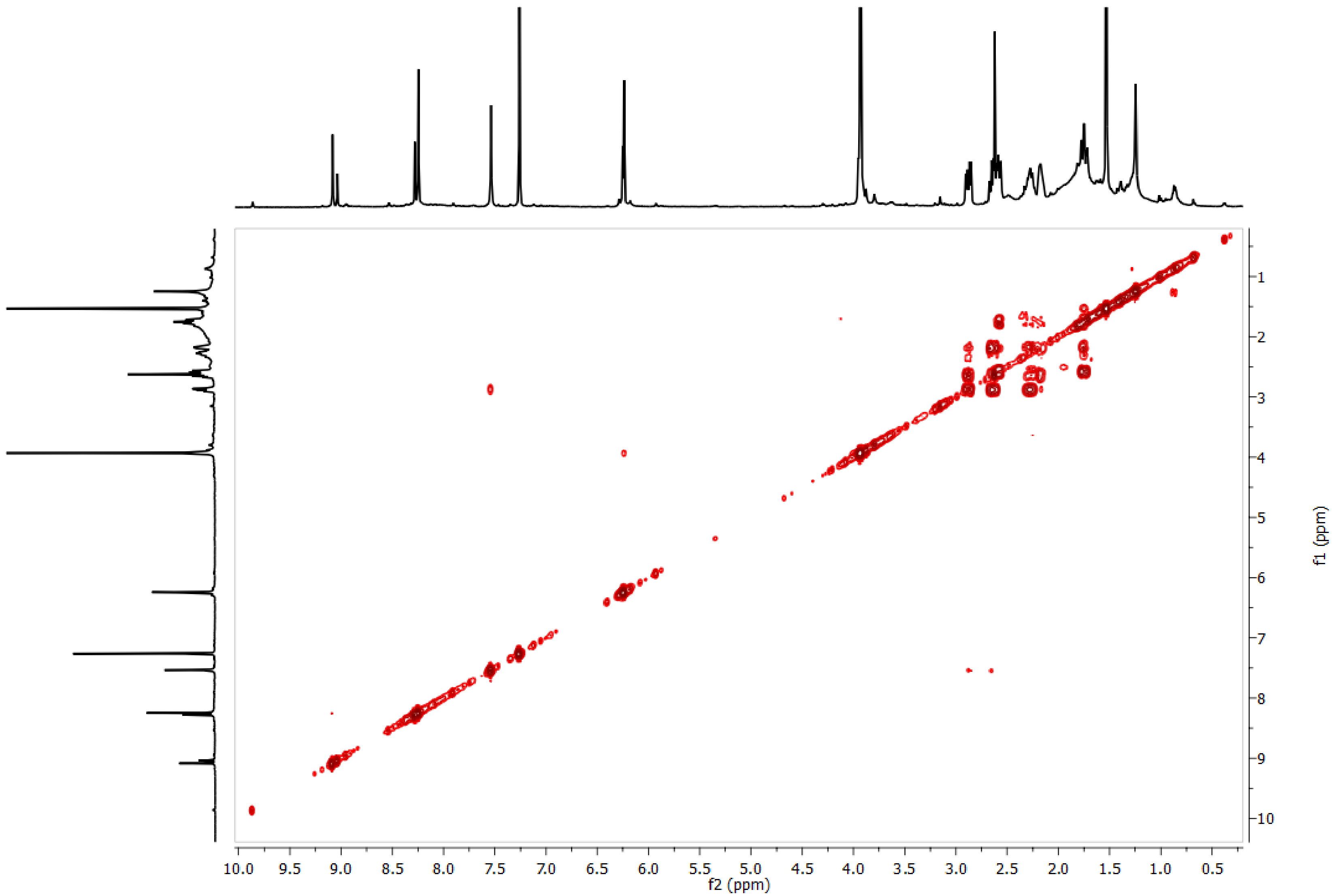

Figure S30. COSY spectrum of 2:1 mixture of 14- and 15-methoxyxestoquinone (**6**) in $CDCl_3$ (500 MHz)

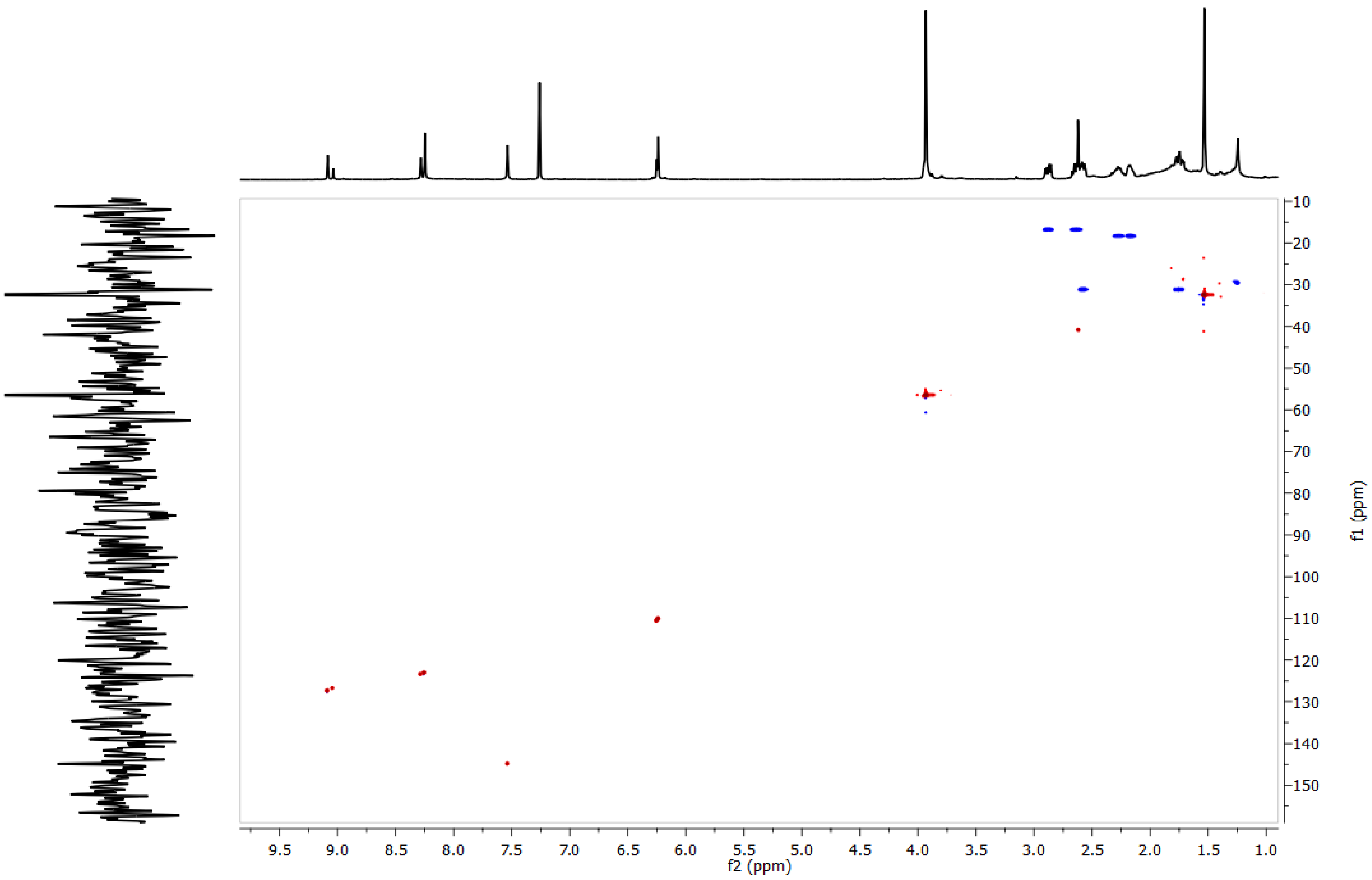

Figure S31. HSQC spectrum of 2:1 mixture of 14- and 15-methoxyxestoquinone (**6**) in $CDCl_3$ (500 MHz)

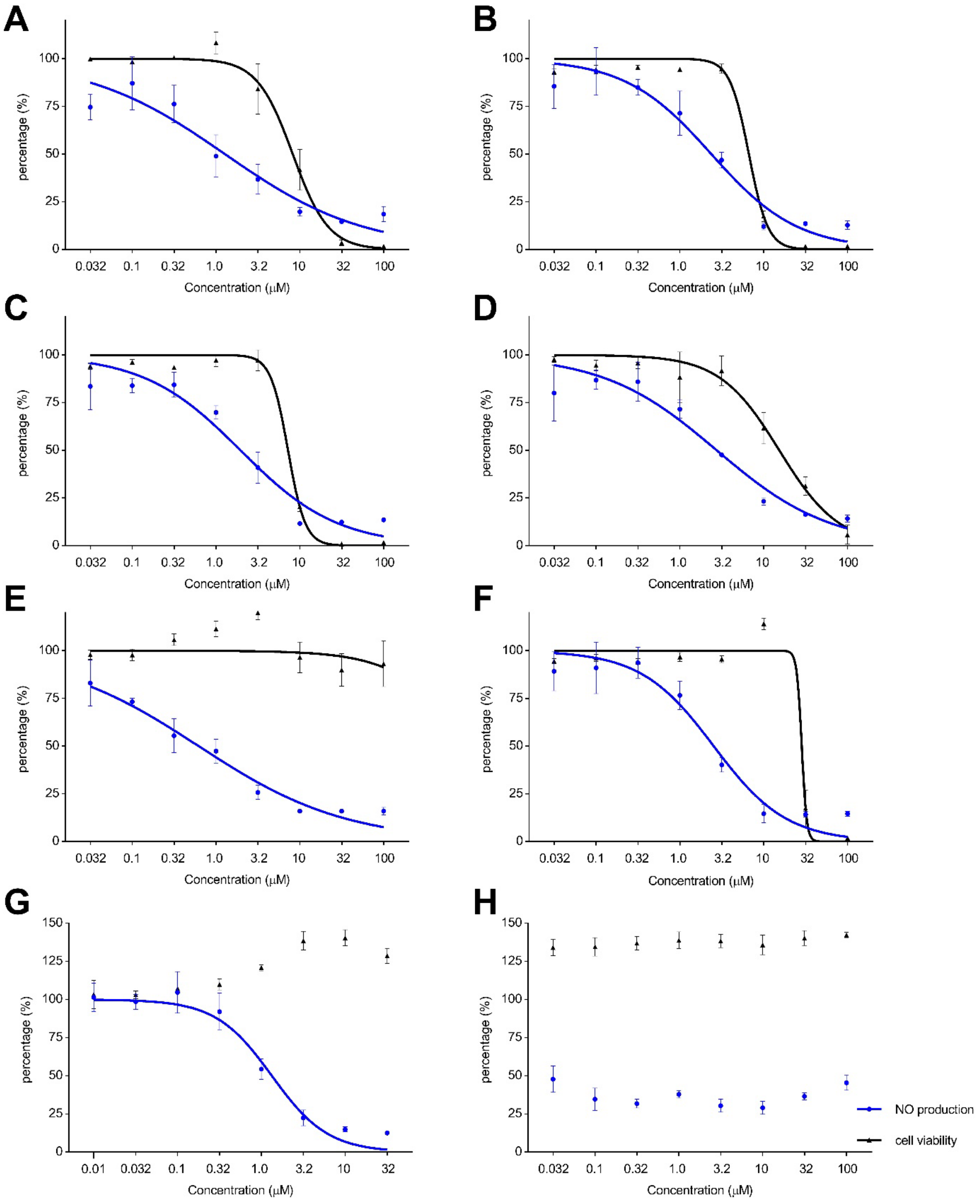

Figure S32. NO production and cell viability of LPS-stimulated RAW264.7 macrophage cells (mean ± SD, n = 3) after pre-treatment with **A**. xestoquinone (**1**), **B**. adociaquinone B (**2**), **C**. adociaquinone A (**3**), **D**. 14-hydroxymethylxestoquinone (**4**), **E**. 15-hydroxymethylxestoquinone (**5**), **F**. 2:1 14-methoxyxestoquinone and 15-methoxyxestoquinone (**6**), **G**. *t*BHQ, and **H**. dexamethasone for 1 h followed by the addition of LPS (Experiment 1).

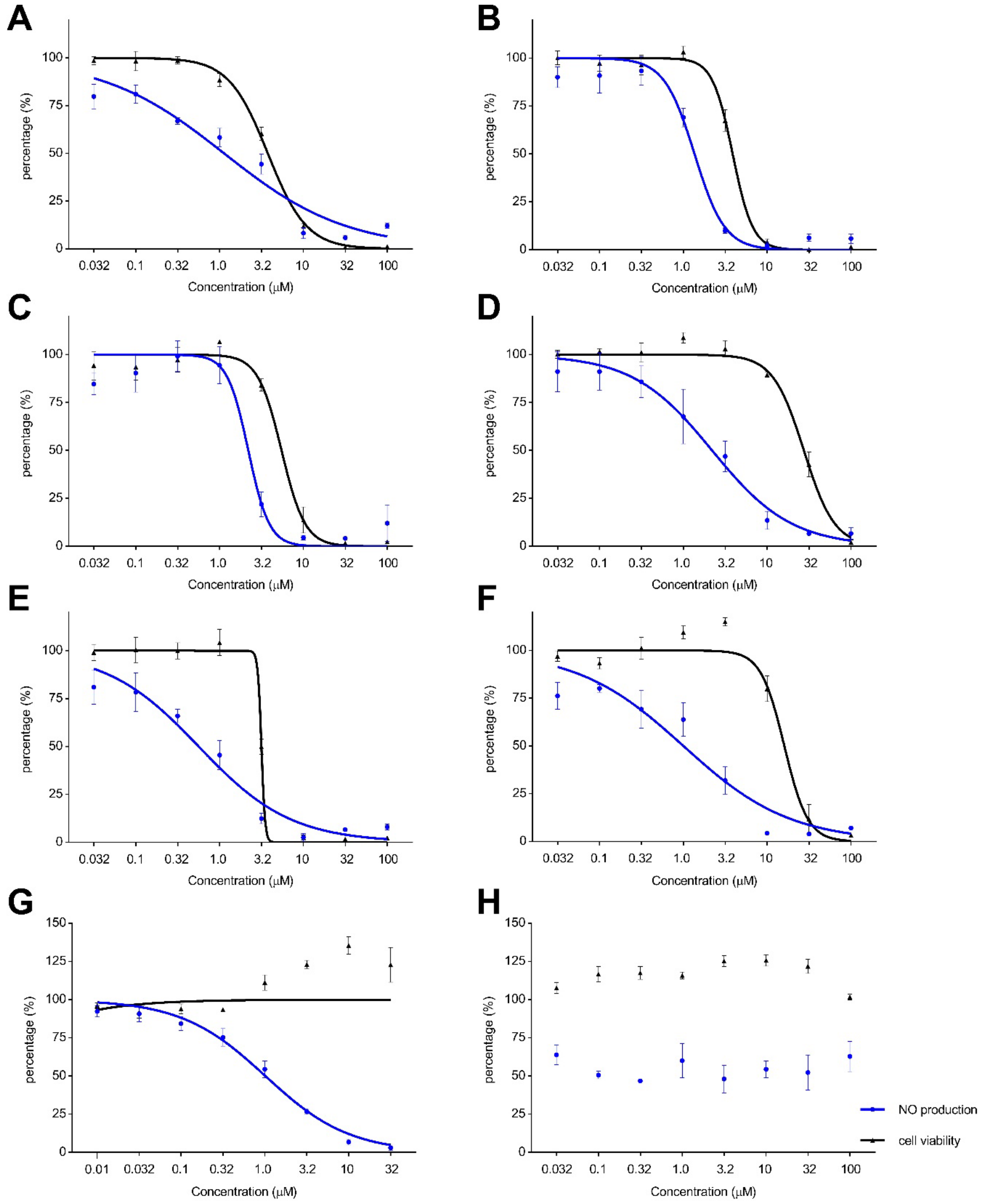

Figure S33. NO production and cell viability of LPS-stimulated RAW264.7 macrophage cells (mean ± SD, n = 3) after pre-treatment with **A**. xestoquinone (**1**), **B**. adociaquinone B (**2**), **C**. adociaquinone A (**3**), **D**. 14-hydroxymethylxestoquinone (**4**), **E**. 15-hydroxymethylxestoquinone (**5**), **F**. 2:1 14-methoxyxestoquinone and 15-methoxyxestoquinone (**6**), **G**. *t*BHQ, and **H**. dexamethasone for 1 h followed by the addition of LPS (Experiment 2).

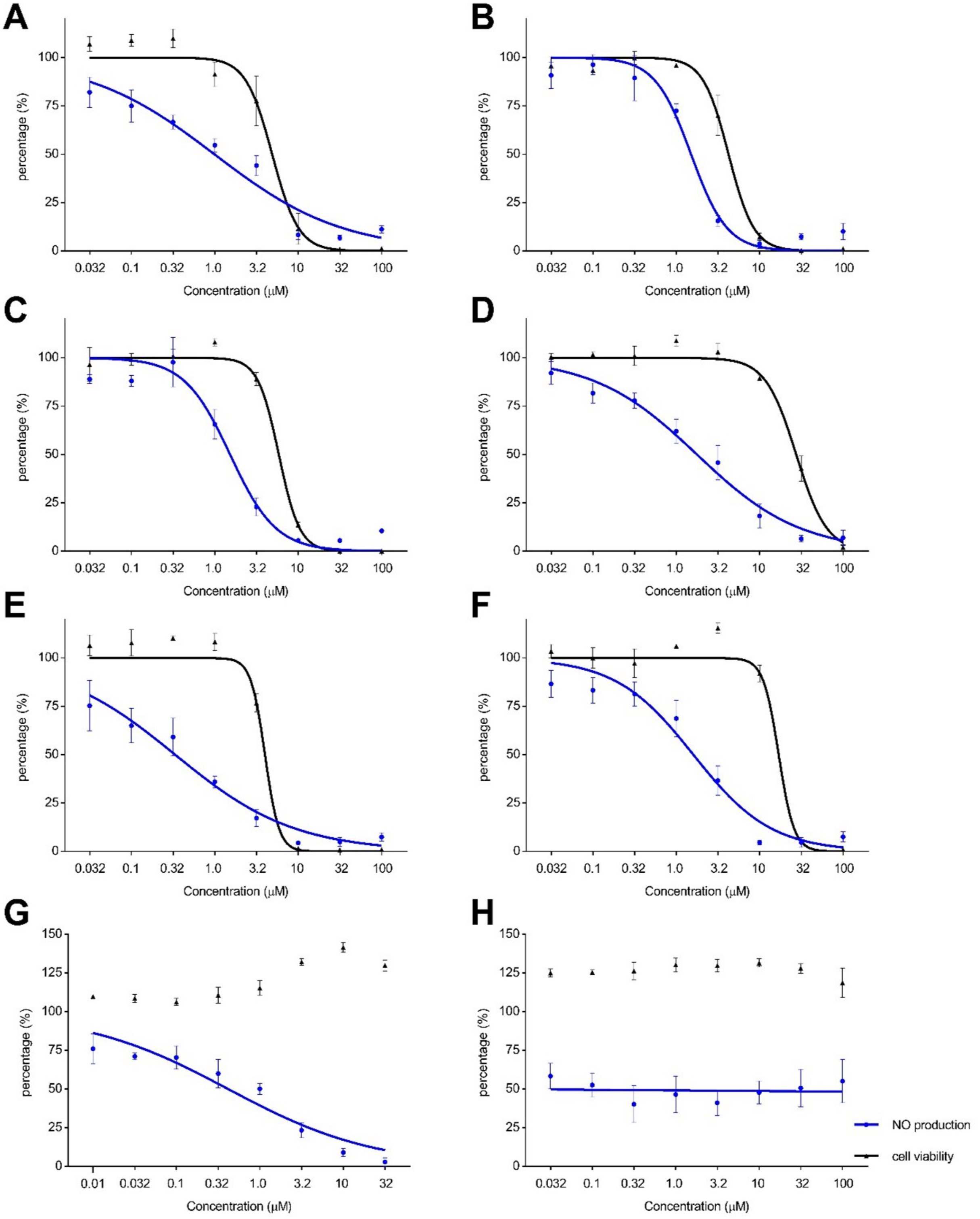

Figure S34. NO production and cell viability of LPS-stimulated RAW264.7 macrophage cells (mean ± SD, n = 3) after using the reverse regimen of pre-treatment with LPS for 1h and followed by the addition of **A**. xestoquinone (**1**), **B**. adociaquinone B (**2**), **C**. adociaquinone A (**3**), **D**. 14-hydroxymethylxestoquinone (**4**), **E**. 15-hydroxymethylxestoquinone (**5**), **F**. 2:1 14-methoxyxestoquinone and 15-methoxyxestoquinone (**6**), **G**. *t*BHQ, and **H**. dexamethasone. (Experiment 1).

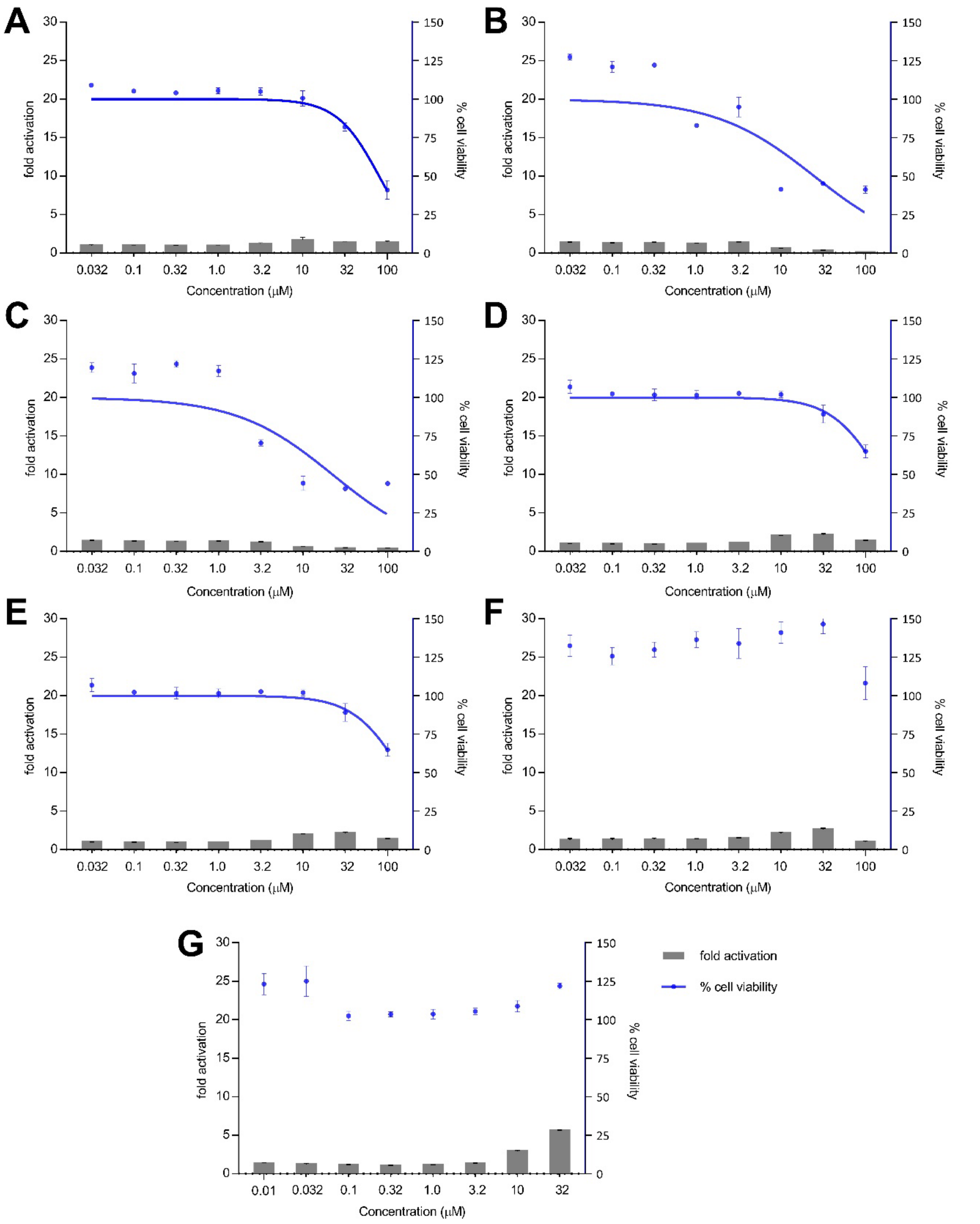

Figure S35. Nrf2-ARE activation (bar graph, data presented as mean + SD, n = 3) and cell viability (line graph, data presented as mean ± SD, n = 3) effects of **A**. xestoquinone (**1**), **B**. adociaquinone B (**2**), **C**. adociaquinone A (**3**), **D**. 14-hydroxymethylxestoquinone (**4**), **E**. 15-hydroxymethylxestoquinone (**5**), **F**. 2:1 14-methoxyxestoquinone and 15-methoxyxestoquinone (**6**), and **G**. *t*BHQ on Nrf2-luciferase reporter MCF7 stable cells after 8 h incubation. (Experiment 1).

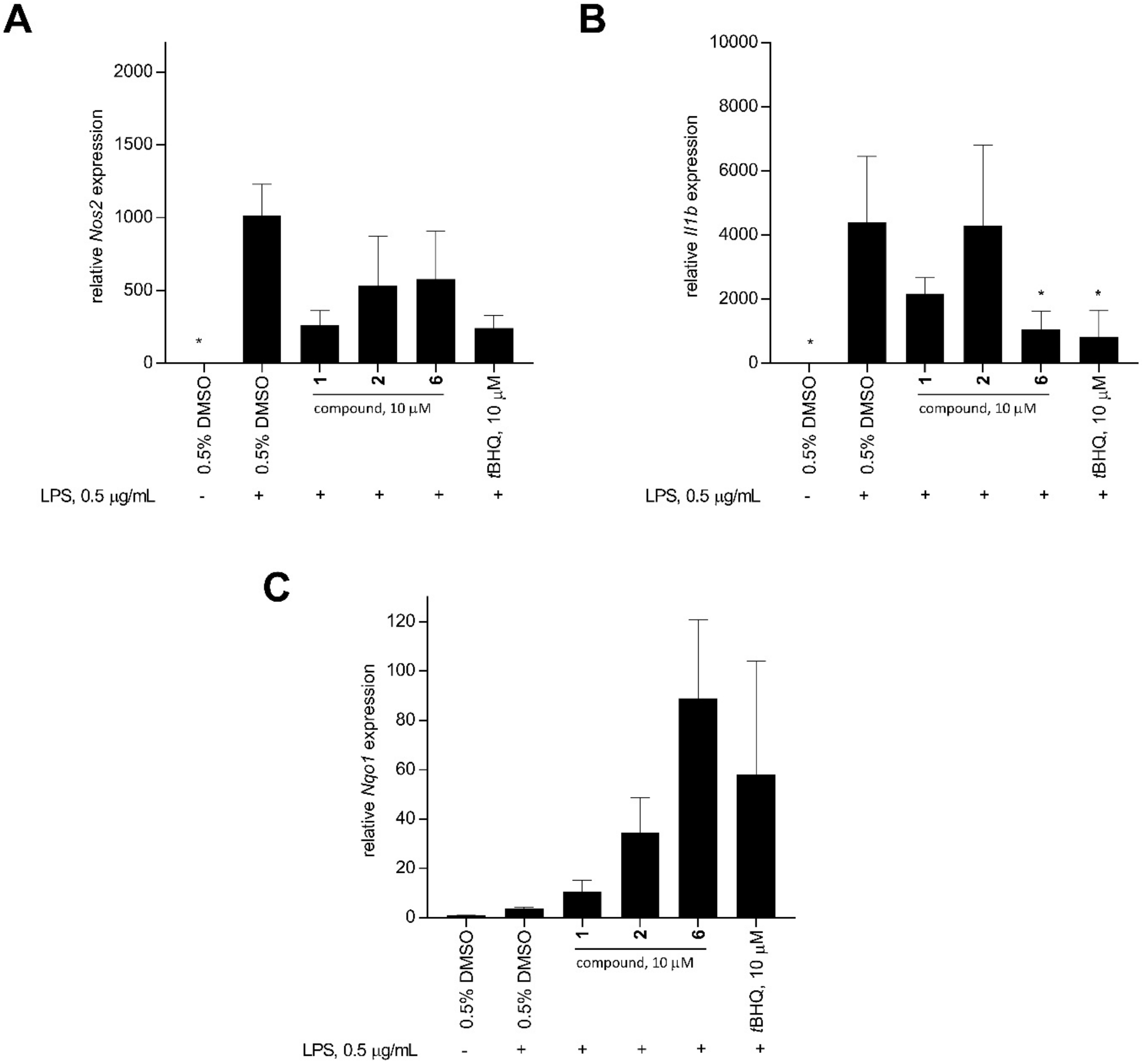

Figure S36. Relative expression (mean + SD, n = 3) of pro-inflammatory and cytoprotective genes: (**A**) *Nos2*, (**B**) *Il1b*, and (**C**) *Nqo1* in LPS-stimulated RAW 264.7 murine macrophage cells after 12-h treatment with 10 μM of xestoquinone (**1**), adociaquinone B (**2**), 2:1 mixture of 14- and 15-methoxyxestoquione (**6**), and *t*BHQ (positive control). Compoubd **5** was not tested due to insufficient material. Mouse *Gapdh* was used as reference gene. Compound **6** showed comparable activity to *t*BHQ in downregulating *Nos2* and *Il1b* expression. Asterisk (*) denotes significant difference relative to 0.5% DMSO + LPS. Data analyzed using one-way ANOVA and Tukey's post-hoc test at p-value < 0.05. (Experiment 2)

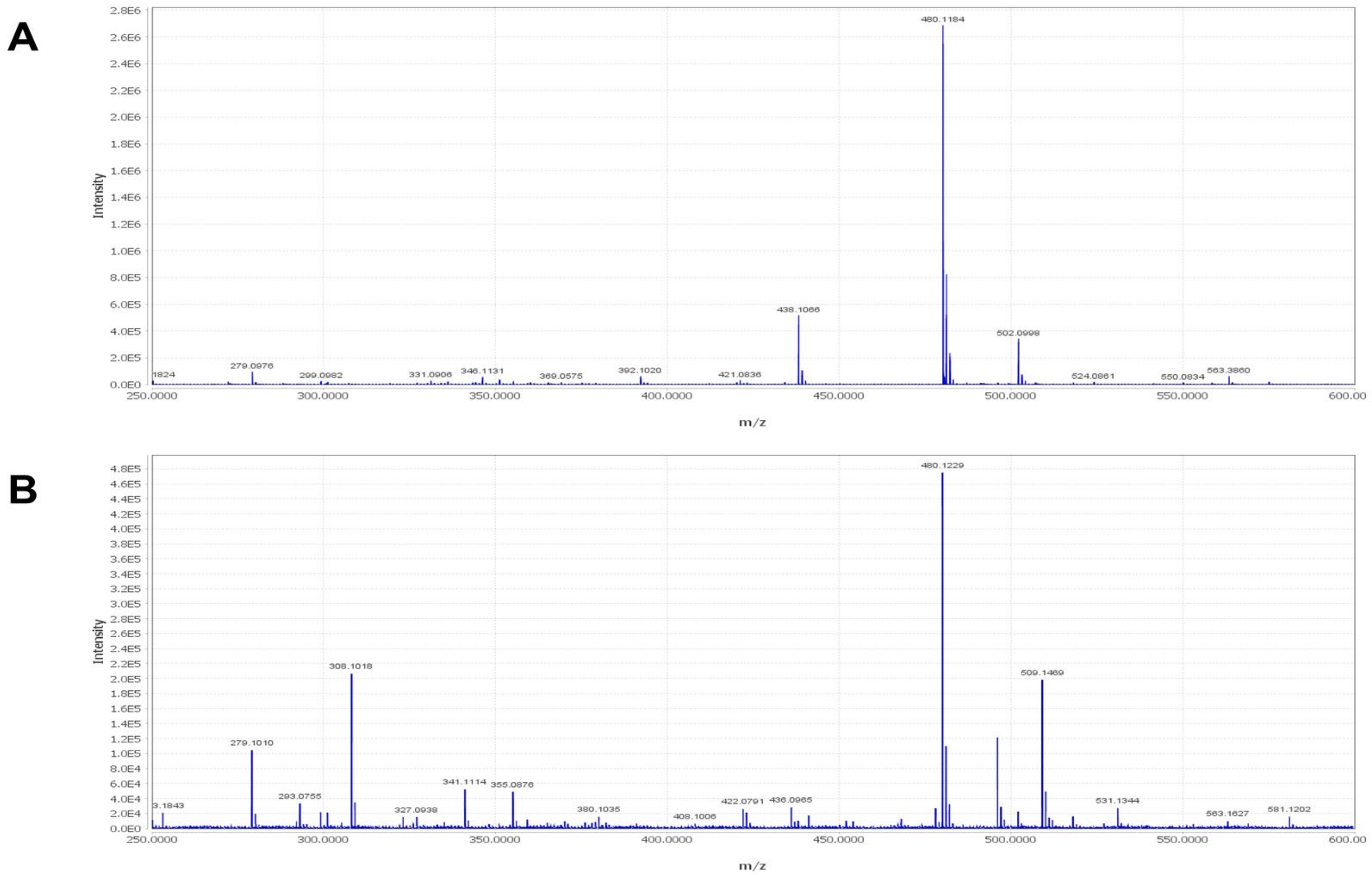

Figure S37. HRESIMS spectra of the reaction of (A) 2:1 and (B) 50:1 *N*-acetyl cysteine (NAC) and xestoquinone (**1**) in positive mode
